# Supplementary material for: Measurable health effects associated with the daylight saving time shift
Source: PLoS Comput Biol. 2020 Jun 8;16(6):e1007927. doi: 10.1371/journal.pcbi.1007927 (PMC7302868; doi:10.1371/journal.pcbi.1007927)
Supplement: S1 Appendix — (PDF) [file pcbi.1007927.s001.pdf]

Supplementary Materials for "Measurable Health Effects Associated with the Daylight Saving Time Shift"

Hanxin Zhang[1,2], Torsten Dahlén[3], Atif Khan[2], Gustaf Edgren[3,4], and Andrey Rzhetsky[1,2,5,*]

[1]Committee on Genetics, Genomics, and Systems Biology, The University of Chicago, Chicago, IL, 60637, US.
[2]Department of Medicine, and Institute of Genomics and Systems Biology, The University of Chicago, Chicago, IL, 60637, US.
[3]Department of Medicine Solna, Clinical Epidemiology Division, Karolinska Institutet, Stockholm, SE-171 76, Sweden.
[4]Department of Cardiology, Södersjukhuset Hospital, Stockholm, Sjukhusbacken 10, 118 83, Sweden.
[5]Department of Human Genetics, The University of Chicago, Chicago, IL, 60637, US.
[*]Correspondence to: andrey.rzhetsky@uchicago.edu

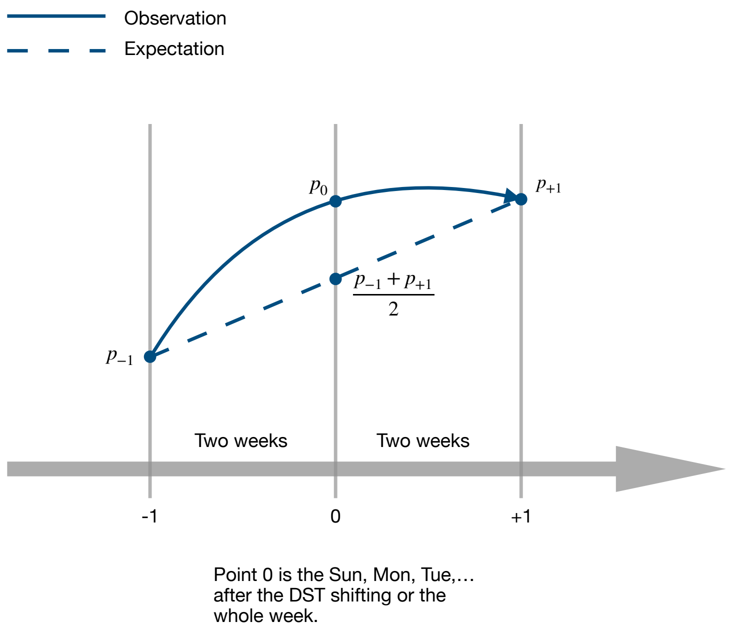

Fig A. $\widehat{RR}$ was evaluated as a ratio of the observed diagnosis rate and the expected diagnosis rate.

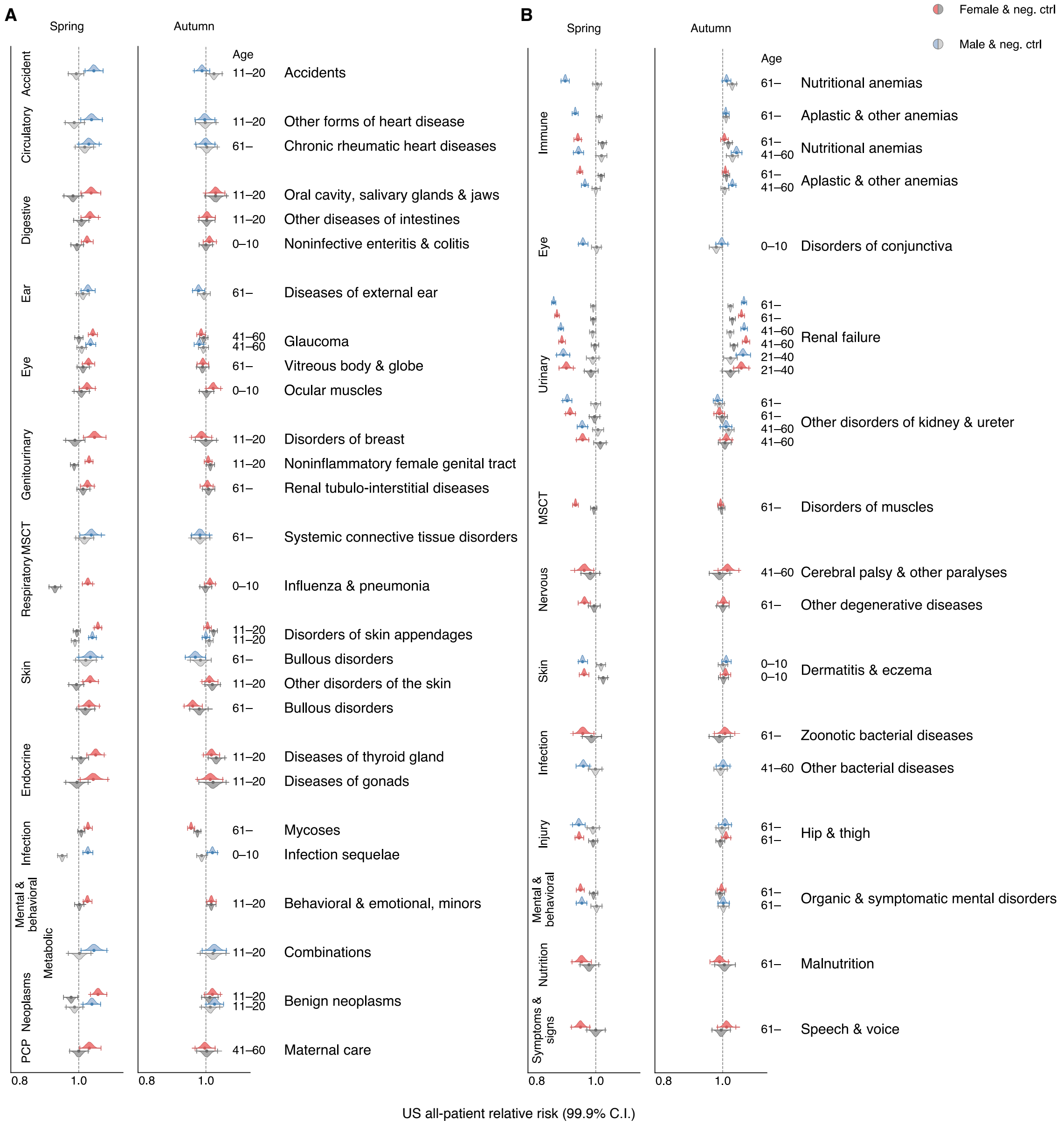

Fig B. The top 30 conditions exhibiting the largest increasing or decreasing risks (effect sizes) for the results of the US all-patient analyses. (A) Increasing signals shown in the US all-patient analysis. (B) Decreasing signals shown in the US all-patient analysis.

1 Materials and Methods

1.1 The MarketScan All-patient Database

The IBM Watson Health MarketScan compiles data from over a hundred large, US-based insurance companies. We used a 2016 snapshot of this database which contained 5,197,121,918 diagnosis records for 151,104,811 unique patients in the US, enrolled from 2003 to mid-2014. For each patient, we knew when and where they entered and left our database. Note that not all patients were enrolled in our database from the start date to the end date. Patients might have been visible for a few weeks, a few months, or several

years. For each diagnosis entry, the database documented the date, the patient's age, and an International Classification of Diseases (IDC) 9th Version, Clinical Modification (ICD-9-CM) code. Because inpatient and outpatient hospitalizations were not discriminated in this database, we call it the "all-patient" database. We have also grouped patients by the state of enrollment; therefore the experimental group includes patients from all states that observe DST, and the negative control group includes patients from states where DST has not been consistently observed (Arizona, Hawaii, and Indiana before 2006). We did not find patients registered in insular territories or minor outlying possessions in the data.

1.2 The MarketScan Inpatient Database

For our analysis, we used an inpatient version of the MarketScan database which documents 496,885,296 inpatient diagnoses records in ICD-9-CM for 175,208,465 unique people from 2003 to 2015. Most patients are duplicates of the enrollees of the all-patient database, but only their inpatient diagnoses were taken into account in this "inpatient database."

1.3 The Swedish Inpatient Registry

The Swedish inpatient database we used incorporates 94,669,631 inpatient diagnoses of 9,419,692 Swedish people from 1968 to 2010. Diagnoses are coded in Sweden's modification of the ICD, 8th, 9th, or 10th versions (ICD-8-SE, ICD-9-SE, or ICD-10-SE). The data are collected from all Swedish hospitals as discharge codes. In the Swedish data, enrollment is nearly static within the weeks surrounding the start or end of daylight saving time. Theoretically, this is because all Swedish inpatient hospitalizations are documented in this database. Unlike the insurance-company-curated MarketScan database, Swedish patients were dis-enrolled only if they died or left Sweden.

1.4 Mappings

The datasets we employed consisted of ICD codes (versions 8, 9, and 10 in the US) and their Swedish modifications. In order to fully utilize these codes, we first created mappings between the different ICD versions. We referred to the CDC General Equivalence Mappings for the translation between ICD-9-CM and ICD-10-CM [46]. We curated the mapping between ICD-9-CM and ICD-8 by ourselves (S14 Table). Additionally, we grouped the ICD-10 diagnosis codes, based on the first three digits, into 263 conditions under 31 systems using the WHO ICD-10 reference (S1 Table) [16]. Neighboring codes tended to fall in the same or related conditions. All ICD-10 codes are categorized in this grouping, so it is also exhaustive. Using all the mappings mentioned above, we produced a UniICD (`ICD_VERSION:ICD_CODE`)-to-condition mapping, and tuned it for both US and Swedish health records in compliance with the difference between US and Swedish ICD modifications in trailing digits (S15-16 Tables).

1.5 Models

We borrowed the idea for the general methodology, and the correction of holidays and day length from a previous study [8]. Thus, we calculated the base diagnosis rate using the expected proportion of patients out of all the enrollees in a certain age group and sex who were recorded as having a specific condition at a specific time point. A time point of interest is a day or week in this study, and it's RR can be quantified as follows:

$$\widehat{RR} = \frac{p_0}{\frac{1}{2}\left(p_{-1} + p_{+1}\right)}, \tag{1}$$

where p_{-1}, p_0, and p_{+1} are diagnoses rates for a particular test group (for example, for a condition in a certain age group and sex, such as diabetes for males aged over 60) at 0, the time point of interest, -1, two weeks before the time point of interest, or +1, two weeks after the time point 0 (Fig A). If some influential

holidays or celebrations fell into the week following the time point −1, or +1, the −1 and +1 points are then adapted to three weeks or one week before and after point 0. In the US data, we considered the following holidays and celebrations: President's Day in February, Western Easter, St. Patrick's Day, Memorial Day, Thanksgiving, Veterans Day, Columbus Day, and Labor Day. In the Swedish data, we considered only Western Easter. In the US, Easter and Thanksgiving showed the largest effect on disease reporting, as we examine in our studies.

The time intervals (called "time points" below) over which we counted disease code incidence, were either day- or week-long. We estimated both day- and week-level RRs for every test group in a Bayesian framework with a hierarchical model. We chose a set of flat, non-informative priors [47] for the diagnosis rates two weeks before and after the day of interest:

$$p_{-1} \sim \text{Beta}(1,1), \tag{2a}$$
$$p_{+1} \sim \text{Beta}(1,1). \tag{2b}$$

In addition, we drew RRs across all test groups from a Gamma prior with the mean μ and the standard deviation σ:

$$RR \sim \text{Gamma}(\text{mean} = \mu, \text{sd} = \sigma), \tag{3}$$

This prior distribution shrank all RRs towards the mean and let information flow across all conditions and test groups. As Gelman *et al.* suggested [17], the multiple comparisons problem is alleviated this way.

Note that all RR estimates (for all disease groups) were sampled simultaneously within the same inference framework; individual estimates constrained each other within one hierarchical model.

Hyper-priors of μ and σ were assumed to be nearly flat:

$$\mu \sim \text{HalfCauchy}(5), \tag{4a}$$
$$\sigma \sim \text{HalfCauchy}(5). \tag{4b}$$

We computed the diagnosis rate on the time point of interest as:

$$p_0 = RR \times \frac{p_{-1} + p_{+1}}{2}. \tag{5}$$

We assumed that the observed incidence values followed binomial distributions:

$$x_{-1} \sim \text{Binomial}(n_{-1}, p_{-1}), \tag{6a}$$
$$x_0 \sim \text{Binomial}(n_0, p_0), \tag{6b}$$
$$x_{+1} \sim \text{Binomial}(n_{+1}, p_{+1}). \tag{6c}$$

in which x_* and n_* ($_*$ is $_{-1}$, $_0$, or $_{+1}$) were observable incidences and the total numbers of enrollees accumulated through years at spring or autumn DST shifts, respectively:

$$x_* = \sum_{y\text{: year of data}} x_{*,y}, \tag{7a}$$
$$n_* = \sum_{y\text{: year of data}} n_{*,y}. \tag{7b}$$

We adjusted for the varied day lengths at a DST shift by multiplying the observed day-level incidences by a factor of either 23/24 or 25/24, for the spring and autumn, respectively.

Finally, we estimated the posterior RR distribution via a Markov chain Monte Carlo (MCMC) sampler (PyMC3) [48] and computed the highest posterior density (HPD) interval as the credible interval. We used the highly efficient No-U-Turn sampler (NUTS) [49], initialized by a variational inference (ADVI, Automatic Differentiation Variational Inference [50]), which generated four independent Markov traces. Each trace was composed of 2,000 tuning iterations and an additional 2,000 drawing steps. Other arguments of NUTS and ADVI such as target acceptance rate, max tree depth, and step scale were PyMC3 3.6's default choices. We computed the Gelman-Rubin convergence diagnostic [51,52] for all RR estimates by comparing the difference between the four traces. The final diagnostic results indicated that all samplings were well-mixed and RR estimates converged rapidly. We have supplied all the Gelman-Rubin statistics we used against the RR estimates in the Supplementary Tables. We computed the final RR estimate distributions based on 2000×4 drawing steps. Again, please note that because the RRs were constrained by an across-the-board, hierarchical prior, we did not need to make formal corrections for multiple tests after sampling.

However, after applying this model to assess the effect of changing to and from DST, we found that, for most test groups, the risk is a little bit smaller than one and the mean of RR is smaller than one. This conflicted with our prior belief that, if DST shifts do not influence health, the relative risk should be approximately one. The less-than-one phenomenon was due to the fact that the disease trend tends to be convex (bent downwards) at the DST shift time points. To compensate for this bias, we corrected the RR using the following equation:

$$\widehat{RR}_{\text{corrected}} = \frac{\widehat{RR}}{\widehat{\mathrm{E}\left[RR\right]}}, \tag{8}$$

In the above, we estimated the RR's expectation $\widehat{\mathrm{E}\left[RR\right]}$ by estimating the Bayesian model's corresponding parameter, μ. This correction ensured that $RR_{\text{corrected}}$'s expectation was one, and for most test groups, the RR was inclined to one. An observed $\mathrm{RR}_{\text{corrected}}$ that was significantly greater than one would mean the upward curvature at the DST shift had exceeded the average natural bent across all test groups. Such a correction procedure is equivalent to initializing μ in Expression (3) to 1.

Because we do not have the enrollment information for Swedish data, that corresponding model was slightly different. The Swedish data was characterized by high coverage and low mobility. Almost all Swedes were visible in the dataset throughout their entire lives. Therefore, we determined it was safe to presume that enrollments did not change from two weeks before to two weeks after DST shifts. We quantified the RR as:

$$\widehat{RR} = \frac{l_0}{\frac{1}{2}\left(l_{-1} + l_{+1}\right)}, \tag{9}$$

where the diagnosis rate $l.$ here is not the proportion but the exact number of incidences expected to be documented for a certain condition at a time point. We still assumed RR to follow a Gamma distribution with an across-all-condition mean μ and we assumed σ as the standard deviation. We set priors for l_{-1}, l_{+1}, μ, and σ to follow a flat, half-Cauchy distribution with a large scale parameter. Again, we assumed the observed incidences to follow Poisson distributions:

$$x_{-1} \sim \mathrm{Poisson}(l_{-1}), \tag{10a}$$

$$x_0 \sim \mathrm{Poisson}(l_0), \tag{10b}$$

$$x_{+1} \sim \mathrm{Poisson}(l_{+1}). \tag{10c}$$

Finally, we accounted for the convex tendency by using Equation 8.

1.6 Alternative Models

As an alternative to the Bayesian method, we also tested frequentist models based on random variables' asymptotic properties. Both types of analysis, Bayesian and frequentist, led to nearly identical conclusions.

Frequentist Method

For large x and n, we used a normal distribution to approximate the error. The normal approximation of the diagnosis rate is as follows:

$$p \dot{\sim} \mathcal{N}\left(\widehat{p}, \sqrt{\frac{\widehat{p}(1-\widehat{p})}{n}}\right), \tag{11}$$

where $\widehat{p} = x/n$ is a realization of random variable $p = X/N$. (Throughout this text, we use notation $\mathcal{N}(\mu, \sigma)$ to denote a normal distribution with mean μ and variance σ^2.)

Using this approximation, we can compute the normally-approximated p_{-1}, p_0, and p_{+1}, corresponding to the diagnosis rate two weeks before, on and after the day or week of interest. The expected diagnosis rate on the time point of interest, $\bar{p}_0 = \frac{1}{2}\left(p_{-1} + p_{+1}\right)$, is also normal:

$$\bar{p}_0 \dot{\sim} \mathcal{N}\left(\frac{1}{2}\left(\widehat{p}_{-1} + \widehat{p}_{+1}\right), \sqrt{\frac{\widehat{p}_{-1}(1-\widehat{p}_{-1})}{4n_{-1}} + \frac{\widehat{p}_{+1}(1-\widehat{p}_{+1})}{4n_{+1}}}\right), \tag{12}$$

Here, we assume p_{-1} and p_{+1} are conditionally independent given a consistent, disease-specific incidence rate. This assumption is generally true if we admit that every incidence is unrelated but only depends on the disease's intrinsic attributes. The RR is $p_0/\bar{p}_0$, which is the ratio of two normal, random variables. This ratio's distribution was discussed by Hinkley in 1969 [53]. Specifically, for two independent, normally-distributed, random variables $X_1 \sim \mathcal{N}(\theta_1, \sigma_1)$, $X_2 \sim \mathcal{N}(\theta_2, \sigma_2)$, the cumulative distribution function $F(w)$ of their ratio $W = X_1/X_2$ can be approximated by

$$F(w) \rightarrow \Phi\left(\frac{\theta_2 w - \theta_1}{\sigma_1 \sigma_2 a(w)}\right) \quad \text{as} \quad \theta_2/\sigma_2 \rightarrow \infty, \tag{13}$$

where $\Phi(\cdot)$ is the cumulative distribution function of the standard normal distribution, and

$$a(w) = \sqrt{\frac{w^2}{\sigma_1^2} + \frac{1}{\sigma_2^2}}. \tag{14}$$

Let

$$\begin{aligned}
\theta_1 &= p_0 = x_0/n_0, \\
\theta_2 &= \bar{p}_0 = \frac{1}{2}\left(\widehat{p}_{-1} + \widehat{p}_{+1}\right) = \frac{1}{2}\left(x_{-1}/n_{-1} + x_{+1}/n_{+1}\right), \\
\sigma_1 &= \sqrt{\frac{\widehat{p}_0(1-\widehat{p}_0)}{n_0}}, \\
\sigma_2 &= \sqrt{\frac{\widehat{p}_{-1}(1-\widehat{p}_{-1})}{4n_{-1}} + \frac{\widehat{p}_{+1}(1-\widehat{p}_{+1})}{4n_{+1}}}.
\end{aligned}$$

The $(1-q) \times 100\%$ confidence interval of $\text{RR} = p_0/\bar{p}_0 = \theta_1/\theta_2$ can be found by solving the equation

$$\frac{\theta_2 w - \theta_1}{\sigma_1 \sigma_2 a(w)} = z, \tag{15}$$

in which z is the $1 - \frac{q}{2}$ or $\frac{q}{2}$ quantile of the standard normal distribution.

Half-Bayesian Method

For a binomial proportion $p = x/n$, we also used a Bayesian method with a Jeffrey's prior, a beta distribution with parameters $\alpha = \beta = 1/2$, to approximate its distribution. The posterior distribution is also a beta with parameters:

$$\beta = \frac{1}{2} + n - x.$$

For large α and β, we may use a normal distribution with mean and variance

$$\begin{aligned} \mu &= \frac{\alpha}{\alpha + \beta}, \\ \alpha &= \frac{1}{2} + x, \\ \sigma^2 &= \frac{\alpha\beta}{(\alpha + \beta)^2(\alpha + \beta + 1)} \end{aligned}$$

as an approximation. Subsequently, the process of estimating the RR and its confidence interval would be identical to what we have discussed below, in the frequentist method section.

Frequentist Method for Swedish Data

Due to the consistency of enrollment, the frequentist model is simpler for the Swedish data. Again, we assumed Poisson distributions to observe diagnoses as done in Expression 10. Notice that the sum of two independent Poisson random variables is still Poisson:

$$x_{-1} + x_{+1} = x_s \sim \text{Poisson}(l_s = l_{-1} + l_{+1}). \tag{16}$$

We considered the confidence interval for the halved RR:

$$\frac{1}{2} \cdot \text{RR} = \frac{l_0}{l_s}. \tag{17}$$

Ederer and Mantel have shown that the Poisson ratio's confidence interval in the above form can be deduced from the confidence interval of the binomial parameter, $\theta = l_0/(l_0 + l_s)$. This is because x_0, given $x_0 + x_s$, is a conditional binomial distribution [54].

We used the Wilson score interval [55] for θ. The lower and upper endpoints of the confidence interval of RR are as follows:

$$R_L = \frac{2\theta_L}{1 - \theta_L}, \tag{18a}$$

$$R_U = \frac{2\theta_U}{1 - \theta_U}, \tag{18b}$$

where θ_L and θ_U are the lower and upper limits of the Wilson score interval of θ.

1.7 The Multiple Comparisons Problem

The multiplicity involved in constructing thousands of credible or confidence intervals simultaneously in the present study may have given rise to erroneous inferences. We controlled this problem in different–but appropriate–ways for the Bayesian and frequentist (and half-Bayesian) methods.

For the Bayesian method, we controlled multiplicity by an across-the-board prior (Expression 3). We parameterized the prior by using the mean and standard deviation of Gamma but not the commonly-used shape α and rate β. Nevertheless, for RRs in our study, the mean μ is usually close to one and for $\sigma \ll \mu$, the shape and rate $\alpha = \mu^2/\sigma^2, \beta = \mu/\sigma^2$ are large, leading to approximately normal distribution. Sampling for such a prior would shrink all the RRs towards the mean and restrain the significance of posterior intervals. We determined how closely we controlled the multiplicity by using the σ scale. A smaller σ would induce more conservative (wider) posterior credible intervals. In our study, σ was naturally inferred from the whole data pool with a nearly-flat prior.

We adjusted the simultaneous confidence intervals constructed by the frequentist analyses by controlling the false coverage rate (or false coverage-statement rate, FCR) [18]. Benjamini and Yekutieli [18] proposed a very simple procedure to control FCR: $\leq q$, through adjusting the significance level q:

$$q \longrightarrow q \cdot \frac{s}{n}, \tag{19}$$

where s is the number of selected estimates among all candidates, and n is the number of candidates. The selection procedure in our study consisted of simply choosing those RR estimates significantly less or greater than one, meaning the **unadjusted** confidence intervals before any correction procedure covered one. The number of candidates are the total number of RRs we were trying to estimate. It is worth noting that we applied these correction procedures only to the selected estimates, whose unadjusted confidence intervals do not cover zero, as suggested by Benjamini and Yekutieli in the original article [18]. Other insignificant confidence intervals remain unchanged. This type of correction procedure ensures that the FCR, i.e., the proportion of the true parameter's failure coverage rate, is less than or equal to q, among all selected intervals.

1.8 Negative Controls

We implemented negative controls in a few ways. For the Swedish data, we analyzed records before 1980, when the DST shift was not obeyed, as a negative control. First, we calculated the pseudo-DST shifts' RRs from 1968 to 1979. We determined the pseudo-DST shift using the following equation:

$$\text{Pseudo-date of DST shift in year } y = \text{Date of DST shift in year } (y+12) - 12 \times 365 - 2, \tag{20}$$

where $1968 \leq y \leq 1979$. We deducted two days in this equation to calibrate the pseudo-DST dates to Sundays. We compared these pre-DST-shift results to the results of the RR estimation for data after 1980. Because we applied the intervention to the entire population of Sweden (comparing patients to themselves before and after intervention), we treated Swedish data as a natural experiment and deduced a stronger conclusion.

All the US health data were collected after the DST shift policy was implemented, so we were unable to contrast results for actual DST shifts with no-treatment control observations (observing DST shift dates, but no DST shift, as we were able to do with Swedish data). We therefore designed negative controls in two different ways:

1. We used health statistics from US states that do not observe the DST shift (Arizona, Hawaii, and Indiana before 2006) as a negative control. However, we found the population too small to inform any statistically reliable conclusions. Compared to the experimental group, which included hundreds of millions of patients who observe the DST shift, this control group only consisted of a few million people. For many diseases, we only have less than ten incidences a day for a specific age-sex stratification.

2. We applied our pipeline to dates other than those with DST shifts. For spring, we repeated the whole analysis for 28 days after the DST shift (day and week). For autumn, the control date was selected at 28 days

before the DST. Again, we adjusted for holidays: We avoided President's Day in February, Western Easter, St. Patrick's Day, Memorial Day, Thanksgiving, Veterans Day, Columbus Day, and Labor Day. Similar to the previous Swedish experiment in 1980, we addressed this negative control as "pseudo-DST" analyses on other dates.

1.9 Geographic Location and Other Covariates

Geographic location will drive variance in daytime length and therefore may affect the DST shift's influence on health. Thus, we divided the applicable states that observe the DST shift into two groups (northern and southern) parts and performed separate analyses. The north part includes Oregon, Idaho, Wyoming, Nebraska, Iowa, Illinois, Indiana, Ohio, Pennsylvania, New Jersey, and states on the north side of the above-mentioned states' south border. The south part includes states on the south side of the northern states' south border.

We also considered another covariate: the difference in culture and work-life balance between western and (north) eastern states. The west part includes Washington, Oregon, California, and Nevada. The east part includes Maine, New Hampshire, Vermont, Massachusetts, Connecticut, Rhode Island, New York, Pennsylvania, New Jersey, Maryland, Delaware, Virginia, and the District of Columbia. We performed analyses separately for these two areas.

1.10 First-time Diagnoses

The potential dissimilarity between the effects of DST shifts on a condition's first incidence and a recurrent follow-up diagnoses is also noteworthy. Because of most patients' incomplete enrollment in the MarketScan data, we were not able to extract a condition's first diagnoses. We had the entire medical history from birth of only a small proportion of zero to ten-year old children (around six million), and, in those cases, we were able to extract first diagnoses. We performed analyses on these patients combining all the DST shift states, along with a negative control experiment on "pseudo-DST" shift dates. A negative control on the non-DST states (Arizona, Hawaii, and Indiana before 2006) was unfeasible due to lack of data.

For completeness and comparison purposes, we still performed another analysis on a disease's first-time diagnosis in each patient's insurance claim sequence in the MarketScan data (US all patients). This first observable diagnosis is not necessarily the true first incidence of a disease, but could be useful for comparison.

As the Swedish register has the complete hospitalization profile for all patients, it allows us to distinguish the first-time diagnoses of chronic disorders from follow-up visit diagnoses. Thus, we also performed tests for all Swedish inpatients. However, because the reduced data provided a much smaller sample size (lifetime first-time diagnoses only, for everyone), this prevented our signals from reaching statistical significance using either the Bayesian or the frequentist method. Note that the Swedish dataset only contains inpatient diagnoses, so we are not able to know if there were any identical but non-hospitalized diagnoses occurring before the first inpatient records of a particular condition.

1.11 Methodological Limitations

The fundamental assumption of the model, illustrated in Fig A, is that the short-term trend of a disease incidence is approximately linear for the weeks surrounding a DST shift. If this is true, we would be able to detect irregular disease incident variation by comparing the observed versus the expected (average) diagnosis rates. This assumption might be violated for some highly seasonal and fluctuating conditions. We implemented the negative controls and validated the results in two countries to alleviate this issue. We are comfortable interpreting only DST shift signals supported by the results of statistical tests of various types across two countries. The set of US-only signals has to be interpreted more carefully: While results are "real" in the statistical sense, very large datasets capture a plethora of various signals (social, ethnic, economic, cultural, and climate- and weather-related) that do not lend themselves to easy deconvolution.

1.12 Data Limitations

Because we focused solely on diagnostic code use around DST shift dates, the data we used in this study were not large enough for some rare diseases. In addition, because many people's insurance enrollment intervals do not overlap with the DST shift dates, the US insurance claim data only covered around one-tenth of the US population (35 million) during and around those dates. For some rare conditions, we observed only a few instances in both datasets (the US and Swedish), so the statistical power for detecting putative DST shift signals was insufficient.

It is possible that disease coding errors could influence our RR estimates. A simple way to spot miscoding is to look for sex-specific codes assigned to the wrong gender. For instance, the data shows males diagnosed with pregnancy, ovarian cancer, and females diagnosed with prostate cancer. There are two scenarios to explain the origins of such errors: Either the sex was recorded wrongly or the code itself is inaccurate. The former scenario would not affect our analyses substantially because we anticipated for symmetric coding errors in both sexes that offset against each other. On the other hand, if the diagnosis itself is miscoded, it may influence the RR estimation and tests. We summarized data for some female- and male-specific diseases as anchor points to estimate the coding error rate (S17-18 Tables). We approximated the error rate using the following equation:

$$\text{(FP + FN) Error Rate} = 100\% \times \frac{2 \times (F_m + M_f)}{F_f + F_m + M_m + M_f} = 0.52\%, \tag{21}$$

where FP stands for "false positive," FN stands for "false negative," F_m is the number of male-assigned, female-specific diagnoses, F_f is the number of female-assigned, female-specific diagnoses, M_m is the number of male-assigned, male-specific diagnoses assigned to females, and M_f is the number of female-specific diagnoses assigned to males.

With our coding error estimates, the error rate is positive, but is small in comparison to the observed DST shift effect sizes.

Importantly, simple disease coding errors are unlikely to be in any way related to DST shifts, and would only bias RR estimates towards the null model.

2 Analyses Summary

All week- and day-level results can be found on the project's web site `https://github.com/hanxinzhang/dst`.

We performed analyses for all 263 disease groups for females and males separately, partitioned into five age groups (0 to 10, 11 to 20, 21 to 40, 41 to 60, and greater than or equal to 61). We started from $263 \times 10 = 2,630$ test groups in total and filtered out those with lower than ten incidences on any day of study. This quality-control step ensured that all the analyzed conditions were statistically meaningful. For sub-common diseases, none of our Bayesian, frequentist, or half-Bayesian methods could give dependable results. Notice that, for the Swedish data, we arranged the age groups slightly differently: 0 to 20, 21 to 40, 41 to 60, and over 60. This difference in analyses groupings does not effect our discussion or conclusions, as we did not find any significant signals in the younger populations in Sweden.

The time periods covered by the study are: (1) the seven days of the week two weeks before a DST shift; (2) the seven days just after q DST shift, and; (3) the seven days of the week two weeks after a DST shift.

For negative controls performed on other dates, including 28 days before or after a DST shift (or pseudo-DST dates before 1980 in Sweden), the days of study were taken around the pseudo-DST dates in a similar way. If any day of study coincided with a holiday or celebration (see 1.5 in the Models Section), we used either one or three weeks before and after the DST or pseudo-DST shift.

The number of test groups and the number of spring week-level RRs that were significantly greater or less than one are summarized in Table A.

Experiment	No. tests	No. sig. (Bayesian)	No. sig. (frequentist)	No. sig. (half-Bayesian)
US all-patient, all DST states	2025	290	265	265
US all-patient, northern DST states	1691	180	287	287
US all-patient, southern DST states	1802	181	191	191
US all-patient, eastern DST states	1471	118	137	137
US all-patient, western DST states	1258	63	81	81
US all-patient, neg. ctrl on other states	546	4	17	17
US all-patient, neg. ctrl on other dates	2041	229	331	331
US inpatient, all DST states	1635	70	64	64
US inpatient, northern DST states	1117	19	28	28
US inpatient, southern DST states	1291	35	35	35
US inpatient, eastern DST states	854	16	8	8
US inpatient, western DST states	633	11	16	15
US inpatient, neg. ctrl on other states	218	3	1	1
US inpatient, neg. ctrl on other dates	1639	28	31	31
Swedish inpatient since 1980	836	7	4	
Swedish inpatient before 1980	242	0	0	

Table A. Summary of Our Analyses

2.1 The Signal Selection Procedure for Presenting the US Results

Conditions with Increased Risk During Spring DST Shifts

We designed multiple controls and compared them to spring DST shift tests to corroborate the risk estimation associated with these DST shifts.

To perform the US analyses, we chose negative-control dates (pseudo-DST) near the actual DST shift dates; in addition, we used US states that do not observe DST ("non-DST states") as negative controls (Section 1.8). Our analyses of Swedish data were somewhat simpler because Sweden did not observe DST before 1980, providing a natural negative control. We present all significant spring signals for the Swedish analyses in the main text (Fig 2B) because all of their natural negative controls before 1980 are not significantly different from one. We used similar selection criteria – by comparing the negative controls to the responses of actual DST shifts – to identify potential conditions associated with spring DST shifts in the US analyses (see the descriptions in the following paragraphs).

Autumn DST shift dates could be viewed as controls for spring's disease RR changes. We did not expect much risk to be associated with the period surrounding autumn DST shifts. A previous study on DST shift association with acute myocardial infarction used solely the autumn tests as control [8]. In contrast to that study's design, we decided to focus on the pseudo-DST shift's negative control near the actual DST shift dates for several reasons.

First, the negative controls we used on the non-DST states did not render a sufficient number of observations; the data was too sparse to allow for a fair comparison to the experimental tests on actual DST shifts. We filtered out many conditions during our quality control step due to their low incidence in the non-DST state data, and it was not even possible to make comparisons for these diseases.

Second, for some diseases, the one-hour disruption – in either direction – seemed to lead to an RR increase. For example, behavioral and emotional disorders in young adults increase after the DST shift in both spring and autumn (Fig B panel A). We might conclude that the autumn DST shift may also have negative effects if this signal were not compared to the negative control tests, which actually revealed a dubious effect in autumn because the negative control test showed a close result.

Third, we accounted for seasonal variation patterns and disease incidence curvature by comparing actual DST time points with "pseudo-DST" time points, both chosen close enough (a few weeks) to the actual DST points. The goal in such an analysis is to account for seasonal confounding factors. Fig B panel A shows the

example mentioned, in which mental and behavioral disorders in autumn seem to increase for both actual DST and pseudo-DST shifts with no differentiation between. This indicates such inflation is more likely to be caused by the trend's upward curvature or disease seasonality as opposed to DST time shifts.

We selected *all* diseases with an increased risk that could be putatively associated with the spring DST shift using the following criteria: (1) The estimated spring DST shift's RR should be significantly greater than one after Bayesian shrinkage or frequentist FCR correction, and; (2) The RR associated with the pseudo-DST shift near the actual spring DST shift dates should not be significantly greater than one after correction for multiple comparisons. The results are shown in S19 Table (US all-patient, Bayesian), S20 Table (US all-patient, frequentist), S21 Table (US inpatient, Bayesian), and S22 Table (US inpatient, frequentist). The half-Bayesian estimates closely followed the frequentist (see Figs C and D), so we focused on comparing the more divergent Bayesian and frequentist results. Fig E (US all-patient) and Fig F (US inpatient) plot spring DST shifts' RRs versus the spring negative control's RR, with selected conditions based on the above-mentioned criteria showing increased risk in blue. The top five conditions with the largest absolute effect sizes ($\widehat{RR} - 1$) are text-labeled.

Using the above-mentioned selection procedure in conjunction with the Bayesian method, we found 82 increased signals for the US all-patient analysis (S19 Table and Fig E panel A). We found 69 when using the frequentist method (S20 Table and Fig E panel B). Inspecting these results, we noticed a number of ill-defined clinical and laboratory findings, examinations, and health services, for which the increased risk could be attributed to various diseases. In addition, some infections, and possibly infection-related eye, ear, genitourinary, and respiratory diseases showed increased risk. The results also suggest possible inflated risk in some circulatory, digestive, metabolic, endocrine, nutritional, musculoskeletal, skin, neoplasm, and mental/behavioral/nervous system diseases, childbirth problems, and injuries in various body sites (S19-20 Tables). Some anemias also stand out, though only in the frequentist results (and not in the more conservative Bayesian results, S20 Table).

For the US inpatient analyses, we selected 42 conditions using the same two selection criteria (formulated above) with a Bayesian analysis (S21 Table and Fig F panel A), and 39 with frequentist estimates (S22 Table and Fig F panel B). Again, we saw infections, genitourinary, and respiratory diseases' risks enlarge. We also saw increased risks in immune, circulatory, digestive, endocrine, metabolic, musculoskeletal, neoplastic, mental/behavioral/nervous system diseases, childbirth problems, and injuries in various body sites (S21-22 Tables).

Conditions with Decreased Risk During Spring DST Shifts

We performed all our analyses bi-directionally (looking for both increases and decreases in disease risk). *A priori* we expected to find as many decreased signals as increased ones (assuming that signals are distributed randomly with mean zero). This is because, if we assume that there is no effect of changing time, the distribution of RR estimates should then be approximately zero-mean normal. Thus, we focused here on significantly decreased RR signals during the spring DST shift period.

Using a similar positive signal selection, but a reverse procedure, we selected conditions with a spring RR of significantly less than one and a negative control RR not significantly less than one. We summarized these selected conditions in S23 Table (US all-patient, Bayesian), S24 Table (US all-patient, frequentist), S25 Table (US inpatient, Bayesian), and S26 Table (US inpatient, frequentist). Fig G (US all-patient) and Fig H (US inpatient) show the spring DST RRs versus the spring negative-control RRs and selected conditions with decreased risk shown in orange. The top five largest-effect conditions, along with their absolute effect sizes ($1 - \widehat{RR}$), are text-labeled.

Our tests revealed a number of decreased RR signals. The Bayesian all-patient results showed 115 diseases with decreased RR immediately after the actual DST shift, but no significant decrease after the pseudo-DST shift dates (S23 Table and Fig G panel A). When we used the frequentist method, we were able to identify 80 diseases with such behavior (S24 Table and Fig G panel B). We observed that these protective signals were distributed differently with regards to human biological systems–rather than with regards to diseases possessing increased risk. The diseases with significantly decreased RRs are associated with infection, genitourinary/urinary systems, skin, musculoskeletal functions, nervous system, neoplasms,

blood diseases (anemia), and some injuries.

We also found many decreased RR signals in mental and behavioral disorders across various age groups and both sexes in the US all-patient analysis (S23-24 Tables, and Fig B panel B). We observed these signals in disease groups that were very different from those with increased risks.

For instance, organic mental disorders showed decreased RR signals in the senior population, while neurotic, stress-related disorders, and youth behavioral and emotional disorders (including attention-deficit/hyperactivity disorder) showed increased RR signals in the US all-patient dataset. Decreased RRs for circulatory and digestive conditions were also dissimilar from their corresponding, increased RR conditions [This is really unclear. It seems like stating the very obvious that "decreased X" would be dissimilar from "increased X". EG]. Remarkably, cerebrovascular diseases in the middle-aged and senior populations showed decreased RR in the US analysis.

We did not, however, observe any childbirth and pregnancy-related conditions that with decreased RRs in the US all-patient analyses. This observation gives more credibility to one of this study's largest-effect signals – those related to the spring DST shift's increase in disease RR related to maternal care for women of advanced ages.

The inpatient results were generally consistent with the all-patient results (S25-26 Tables, and Fig H). However, some diseases' RR signals reversed signs across age groups. For example, for disease codes associated with "other forms of heart disease, spring DST shift RRs decreased in females aged 41-60 (S25-26 Tables), but increased in some young and senior age groups (S21-22 Tables).

2.2 Methods Comparison

We evaluated DST shift effects on health based on the results of a Bayesian analysis (which had a tendency to be more conservative in terms of the number of signals detected)–though the other methods, Bayesian, frequentist, or half-Bayesian approaches, would have led us to very similar conclusions. Figs C and D show comparisons of the methods we used to estimate disease RR. Each triad – consisting of the blue, red, and purple bars – shows a particular test group's confidence or credible intervals (CI) and RR estimates. All three methods gave us close RR estimates with comparable interval widths, especially for estimates close to one. In those more extreme cases, in terms of effect size, the Bayesian method did provide a smaller RR estimate due to its shrinkage property. The Bayesian method tended to "pull" RRs towards the across-the-board mean if the information from the observation did not surpass the prior. Fig I (US all-patient) and Fig J (US inpatient) make this claim more clear. Figs I panel A and J panel A show a scatter-plot of frequentist versus Bayesian estimates. One can see there are two types of estimates on the plot forming two lines of dots – one diagonal and the other off-diagonal. The diagonal line shows conditions with enough observed incidences that all three methods returned very close RR estimates. By contrast, the off-diagonal line shows that the frequentist method returns more extreme estimates (in terms of absolute effect size), while both Bayesian methods shrink the estimates to the prior, no-effect assumption. The RR estimate distribution, shown in Figs I panel B and Fig J panel B, partially explains why there are as many decreased signals as increased signals. The RR estimates follow a normal distribution with symmetric tails. Again, the Bayesian method shows a conservatively shrunk estimation.

2.3 Geographic Location Comparison

The DST shift's health effects may differ between the southern, northern, eastern, and western areas of the US (Figs K and L, Bayesian estimates). For example, the population appears to suffer more from heart diseases in the south and west (Fig L), but the northern and eastern population may be higher risks for injuries and neurotic and stress-related mental disorders (Fig K). Nevertheless, we opt not to make any inference from these comparisons because too many other covariates should be considered, and a larger population is required to draw any meaningful conclusion.

2.4 Results of the First-diagnoses Analyses

US Children (Aged Zero to Ten) with a Complete Medical History

The results of these analyses can be found on the project's web site `https://github.com/hanxinzhang/dst/tree/master/us_allpatient/results_AllStatesWithDst_trueFirstDiag0-10`. The first-diagnoses analyses show a lack of statistical power. Most conditions were filtered out during the quality control stage. We would not trust much in the results, as many conditions were not even tested, and those analyzed were not constrained adequately because of the small test number. The sporadic signals we found in the all-patient, first-diagnoses experiment are more likely to be due to seasonal convexity or concavity – dermatitis, eczema, respiratory, and various communicable diseases. The only remarkable signal is non-infective enteritis and colitis for female children, going up about 3.4 percent in RR in the spring all-patient data. This is also one of the most important discoveries we observed in other analyses, and it is possibly associated with other, stress-related mental health issues and immune disorders.

US All Patients, Condition's First Incidence in the Insurance Claim Sequence

Again, the first-diagnoses analyses show a lack of statistical power due to data limitation. The only significant signal we replicated is the notable circulatory condition in senior females (other forms of heart diseases in female patients over 60) using the frequentist method. The results can be found on `https://github.com/hanxinzhang/dst/tree/master/us_allpatient/results_AllStatesWithDst_firstDiag`.

Swedish Inpatients, Condition's First Hospitalized Diagnosis

Most of the conditions were filtered out due to their low incidences. There is no significant signal in the results. The results can be found on `https://github.com/hanxinzhang/dst/tree/master/se_inpatient/first%20diag`

2.5 Absolute Human Cost of the Daylight Saving Time Shift

Due to asynchronous enrollments (not every patient joined from the first day of the MarketScan database and left on the last day), our data only covered around one-tenth of the US population (35 million) during and around DST shift dates. We estimated the cost of spring DST shifts in terms of incident elevation (S27-30 Tables). For selected, increasing conditions (see Section 2.1 for the selection procedure), we estimated the incident increase by $(\widehat{RR} - 1)\times$ *# of expected incidences averaging points +1 and -1.* To compute the total number of incidences possibly associated with spring DST shifts, we combined conditions with significantly increased RRs in the spring that were not ruled out by the negative control experiment based on our selection criteria, discussed in Section 2.1.

The RR elevation translates to the following incidences in the first week of a DST shift on average, out of 35 million people:

- 600 more inpatient incidents of other forms of heart disease
- 300 more inpatient incidents of ischemic heart diseases in people over 60
- 500 more behavioral and emotional disorders for eleven to 20-year olds
- 200 more diagnoses of non-infective enteritis and colitis in 21 to 40-year olds

These numbers are all based on our Bayesian estimates (shown in S27 Table for US all-patient and S29 Table for US inpatient). We also approximated costs using the frequentist results where the estimation is larger (S28 Table for US all-patient and S30 Table for US inpatient).

In all, we found that around 15,000 incidences of all kinds on average per year in the first week of a DST shift could be linked to the time change. Considering the coverage rate of our data, 0.15 million disease incidents and conditions could emerge due to DST shifts in the US every year. Globally, there could be 0.88 million more disease incidents during the week after the spring DST shift, every year.

References for the Supplemental Content

46. Centers for Disease Control and Prevention (CDC), Diagnosis code set general equivalence mappings – ICD-10-CM to ICD-9-CM and ICD-9-CM to ICD-10-CM [Internet]. https://www.cdc.gov/nchs/icd/icd10cm.htm

47. Prior Choice Recommendations [Internet]. Wiki for the Stan project on github.com [cited 2020 Apr 29]. Available from: https://github.com/stan-dev/stan/wiki/Prior-Choice-Recommendations.

48. Salvatier J, Wiecki TV, Fonnesbeck C. Probabilistic Programming in Python Using PyMC3. PeerJ Computer Science. 2016 Apr 6;2:e55. doi:10.7717/peerj-cs.55

49. Hoffman MD, Gelman A (2011). The No-U-Turn sampler: Adaptively Setting Path Lengths in Hamiltonianmonte carlo. 1111.4246.

50. Kucukelbir A, Tran D, Ranganath R, Gelman A, Blei DM (2017) Automatic Differentiation Variational Inference. Journal of Machine Learning Research 18: 1-45.

51. Brooks SP, Gelman A (1998) General Methods for Monitoring Convergence of Iterative Simulations. Journal of Computational and Graphical Statistics 7: 434-455.

52. Gelman A, Rubin DB (1992) Inference from Iterative Simulation Using Multiple Sequences. Statist Sci7: 457-472.

53. Hinkley DV. On the Ratio of Two Correlated Normal Random Variables. Biometrika. 1969 Dec 1;56(3):635-9.

54. Ederer, F., 1974. Confidence Limits on the Ratios of two Poisson Variables. Am J Epidemiol, 100, pp.165-167.

55. Wilson EB. Probable Inference, the Law of Succession, and Statistical Inference. Journal of the American Statistical Association. 1927 Jun 1;22(158):209-12.

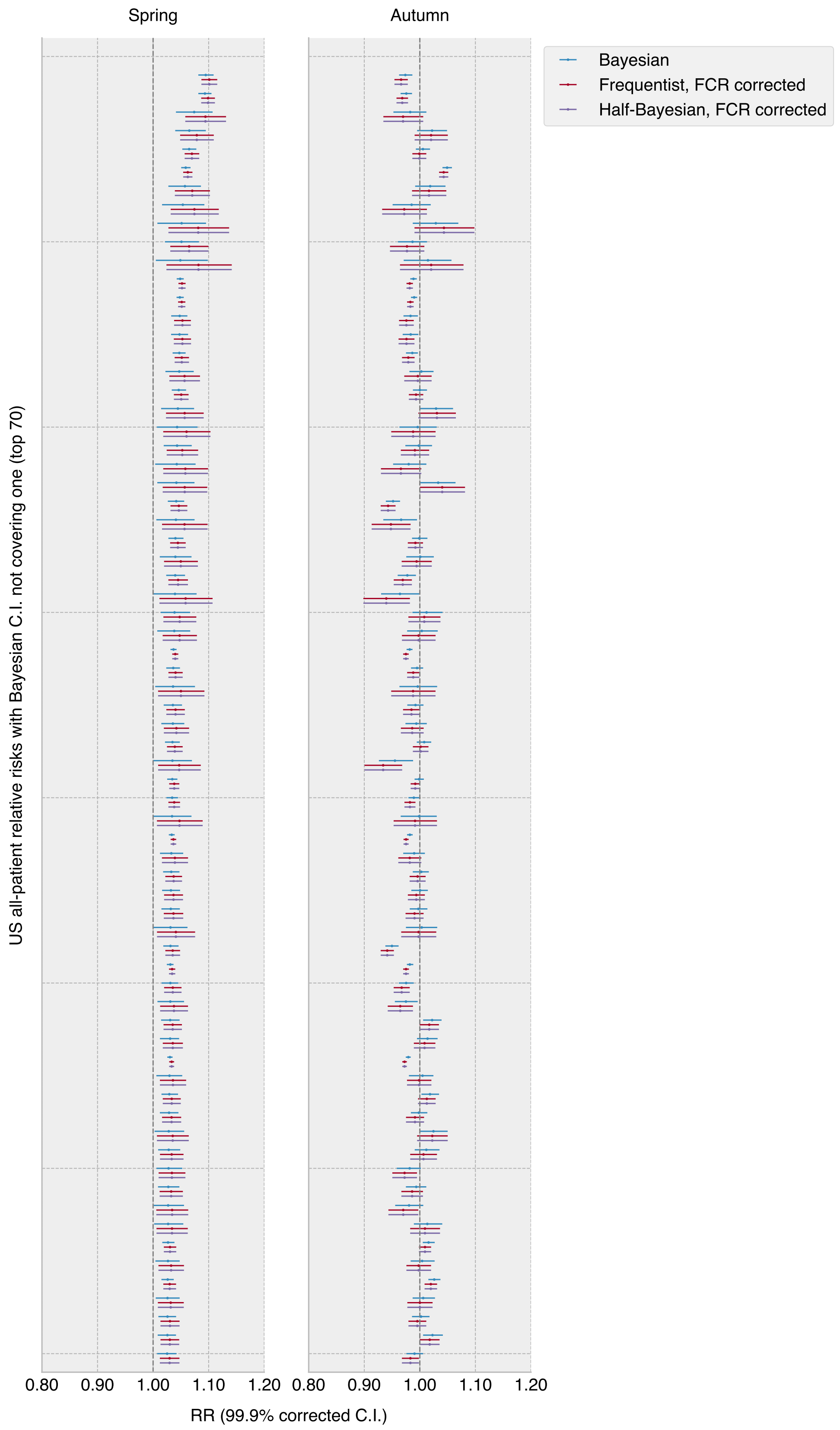

Fig C. Different methods result in similar RR and interval estimates.

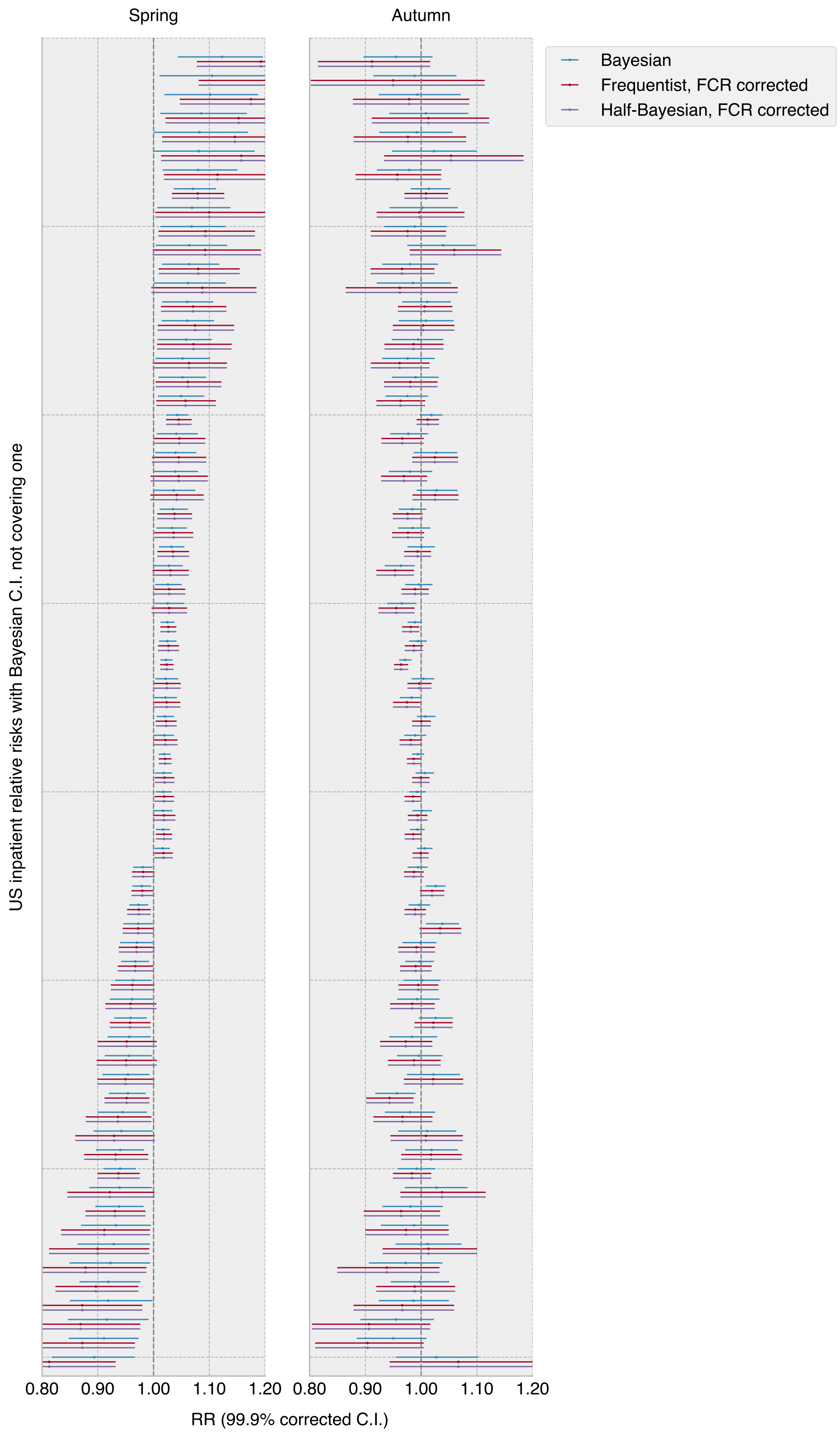

Fig D. Different methods result in similar RR estimates and intervals.

A: Bayesian estimates

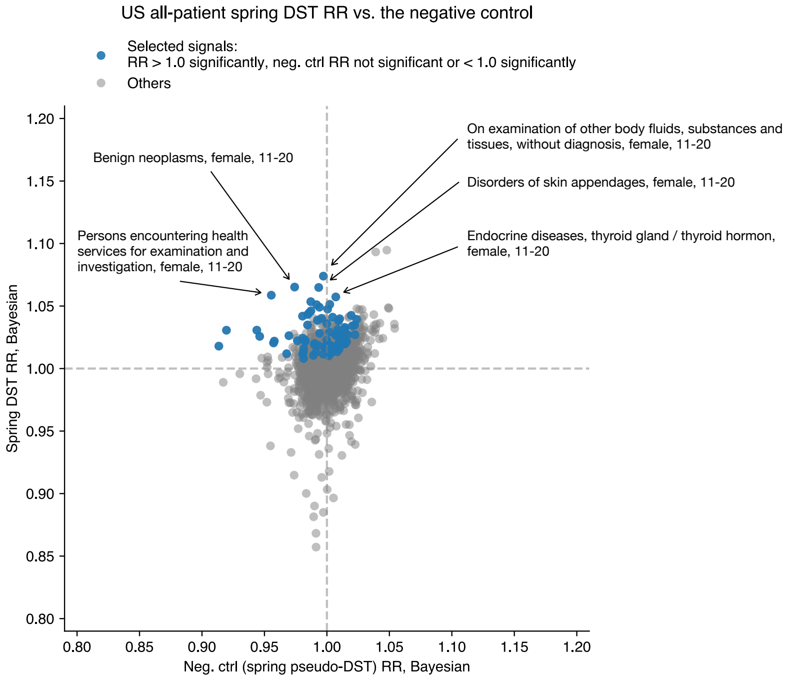

B: Frequentist estimates

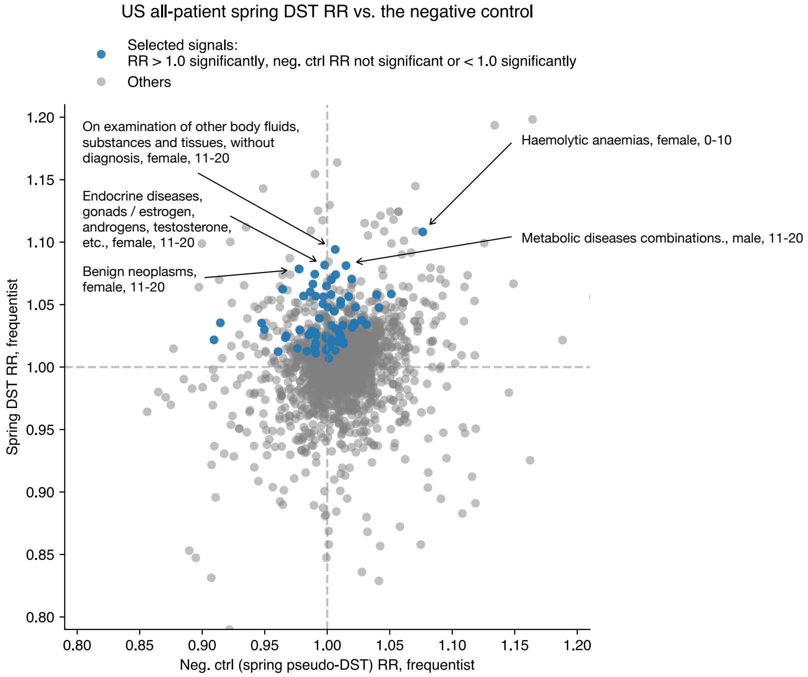

Fig E. Selecting conditions with increased risk by comparing spring DST shift RRs to the negative control on pseudo-DST shift dates. The top five in effect size are annotated. (A) RR estimates generated by the Bayesian method. (B) RR estimates generated by the frequentist method.

A: Bayesian estimates

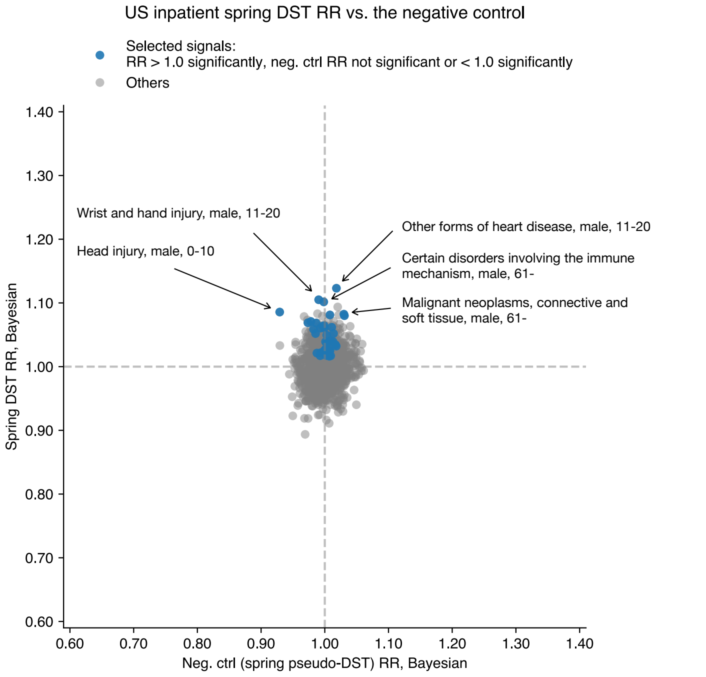

B: Frequentist estimates

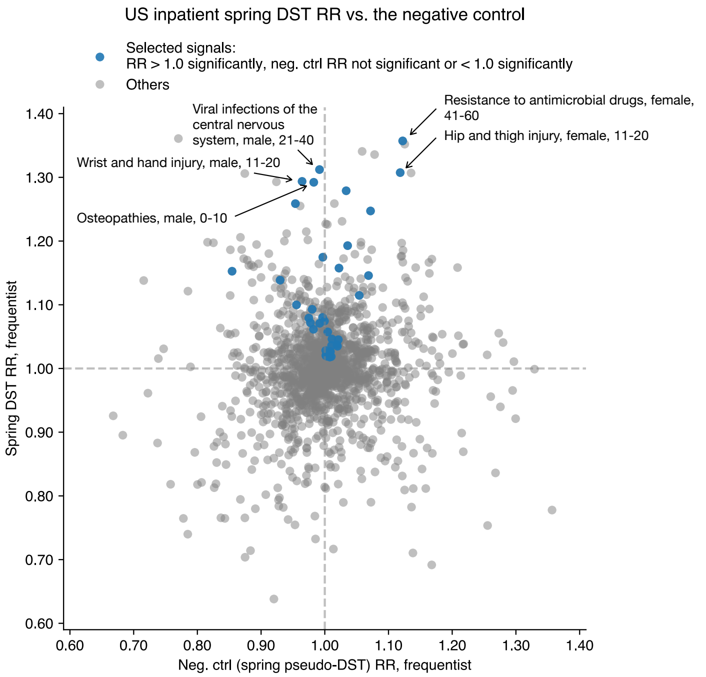

Fig F. Selecting conditions with increased RR by comparing spring DST shift RRs to the negative control on pseudo-DST shift dates. The top five in effect size are annotated. (A) RR estimates generated by the Bayesian method. (B) RR estimates generated by the frequentist method.

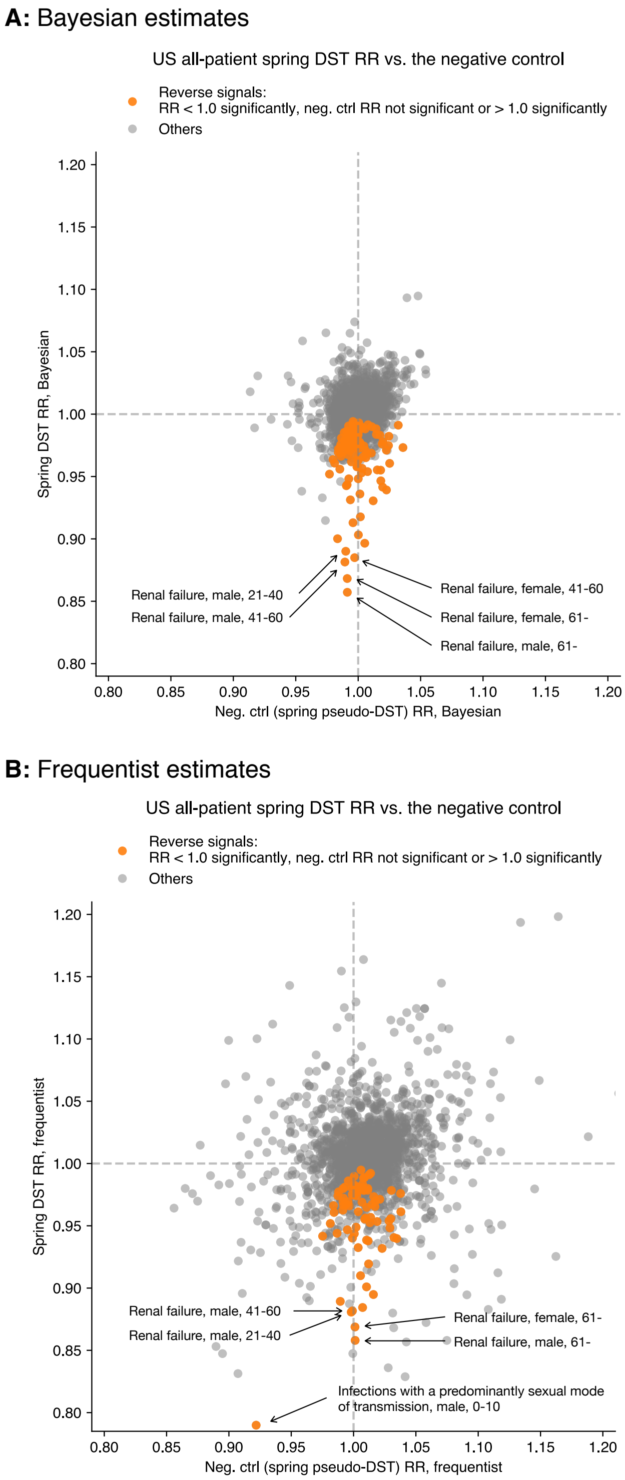

Fig G. Selecting conditions with decreased RR by comparing spring DST shift RRs to the negative control on pseudo-DST shift dates. The top five in effect size are annotated. (A) RR estimates generated by the Bayesian method. (B) RR estimates generated by the frequentist method.

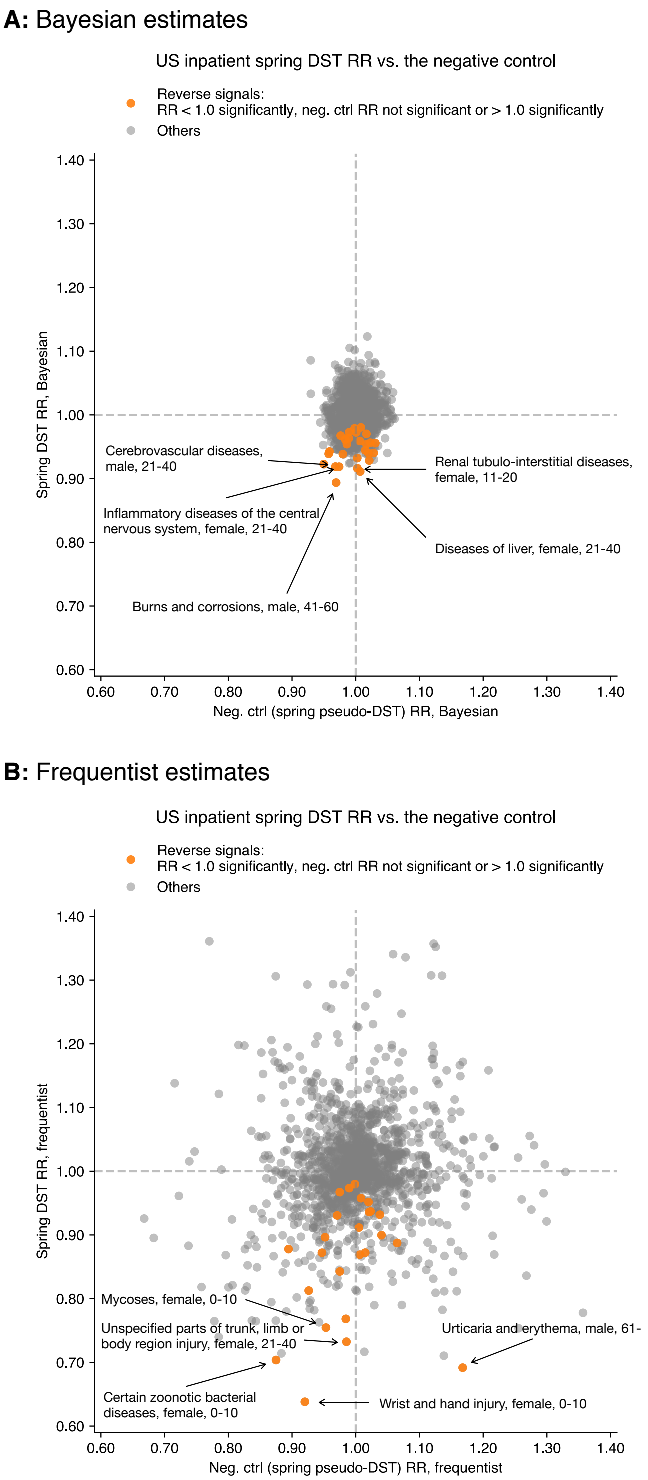

Fig H. Selecting conditions with decreased RR by comparing spring DST shift RRs to the negative control on pseudo-DST shift dates. The top five in effect size are annotated. (A) RR estimates generated by the Bayesian method. (B) RR estimates generated by the frequentist method.

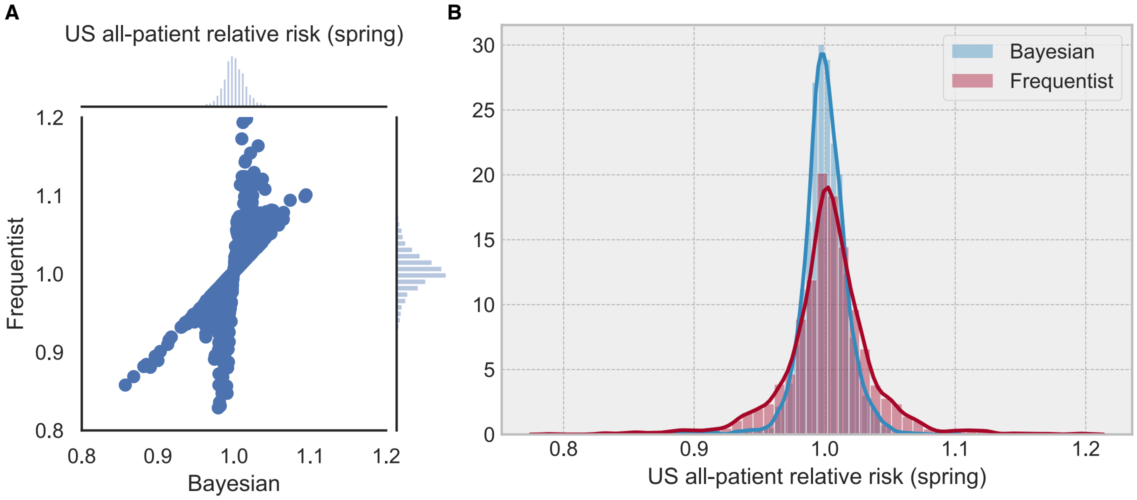

Fig I. The Bayesian method shrinks the estimates to the prior mean when there is not enough information. (A) A scatter-plot of frequentist versus Bayesian estimates. (B) Distributions of RR estimates.

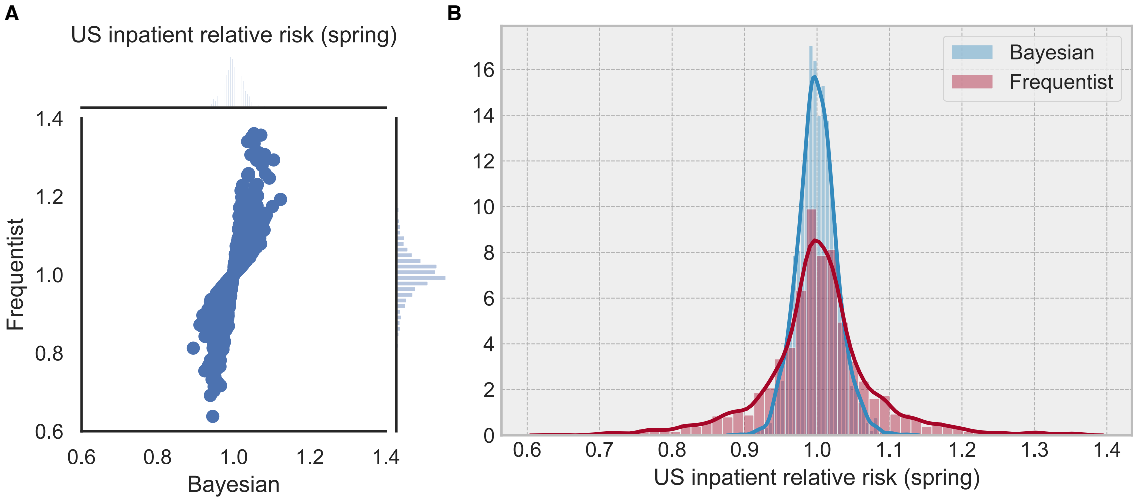

Fig J. The Bayesian method shrinks the estimates to the prior mean when there is not enough information. (A) A scatter-plot of frequentist versus Bayesian estimates. (B) Distributions of RR estimates.

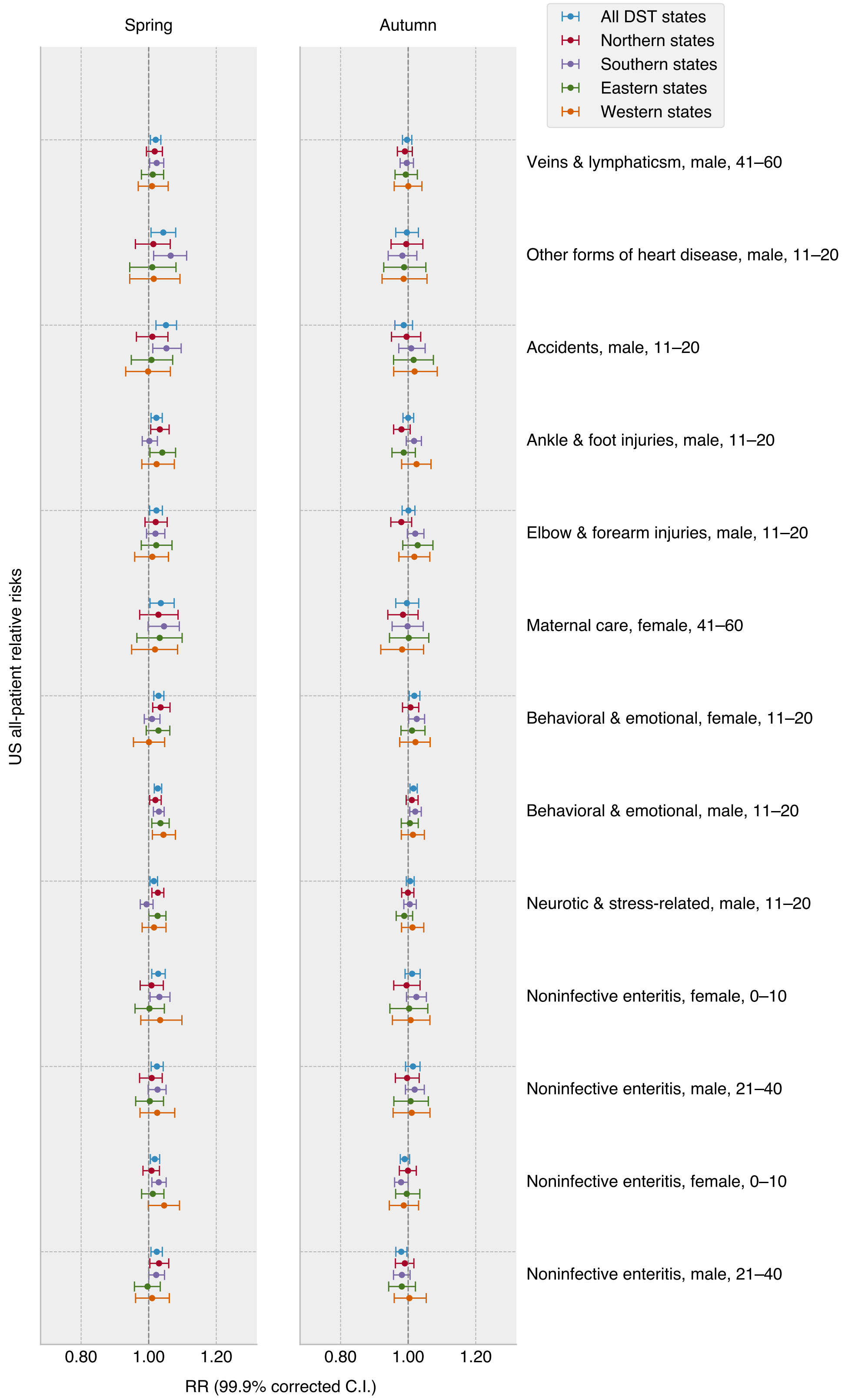

Fig K. The geographic and cultural diversity of the DST shift's effects on health.

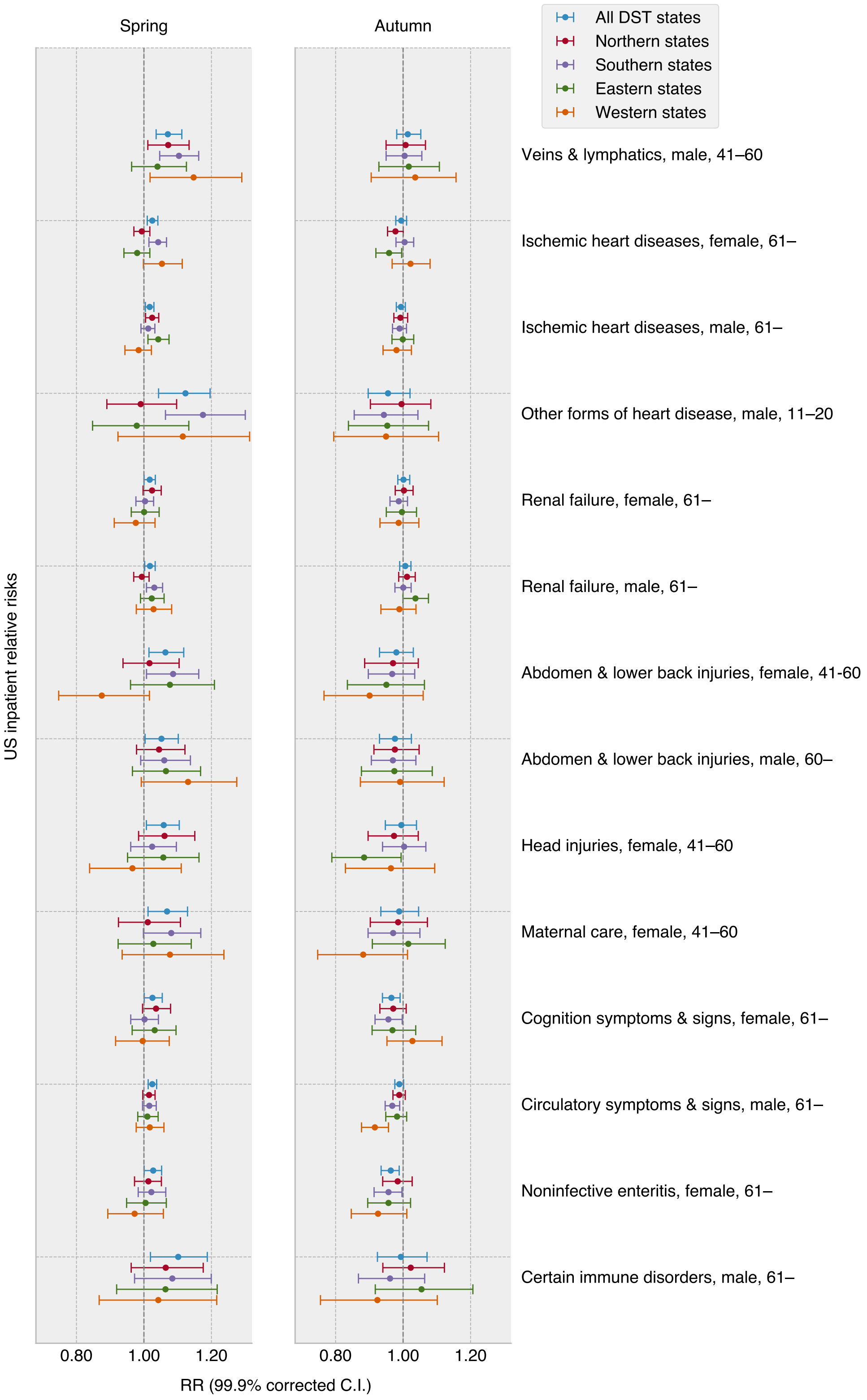

Fig L. The geographic and cultural diversity of the DST shift's effects on health.

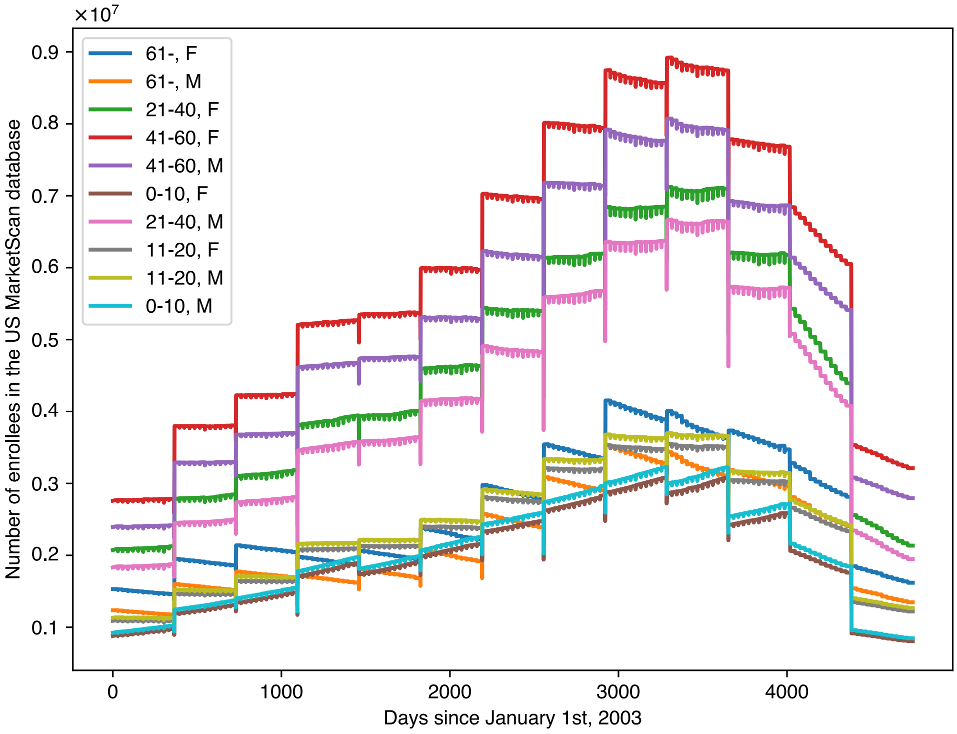

Fig M. Enrollee variation in the US MarketScan data. Integrating the models' real-time enrollee number is an easy step to account for the confounding enrollment variation. Around DST shifts, the total enrollee number does not change much (around one percent on average, see the plot below), but the yearly difference is significant. We observe that the number of enrollees changes several folds (yearly) from 2003 to 2014. The majority of these changes occur at the beginning of each year when new enrollees either joined or left the insurance policy. However, these yearly changes would not significantly affect the RR estimation, as we always compared the incidences from the few weeks around DST shifts.

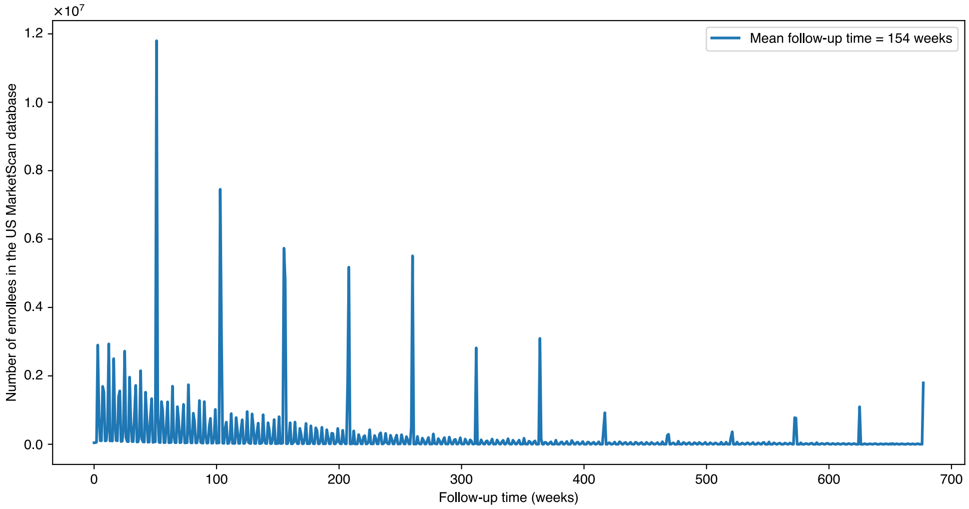

Fig N. Number of enrollees in the MarketScan dataset (Y axis) by week of study (Y axis). Week 0 in this representation starts on January 1, 2003, and the weeks are numbered sequentially after that.
