# Supplementary material for: Measurable health effects associated with the daylight saving time shift
Source: PLoS Comput Biol. 2020 Jun 8;16(6):e1007927. doi: 10.1371/journal.pcbi.1007927 (PMC7302868; doi:10.1371/journal.pcbi.1007927)
Supplement: Attachment — Submitted filename: reply_to_reviewers_V6.docx [file pcbi.1007927.s032.docx]

**Reviewer #1**

The paper presents a large-scale analysis of how daylight-saving time (DST) affects people’s health for a wide range of diseases using EMR data. They use US market scan with around 120 million patients and the Swedish national patient registry with 9 million patients. They use Bayesian inference to estimate the effect size and include a frequentist approach for reference. They reproduce diseases earlier shown to be affected by DST and come with novel results, like pregnancy related diagnoses and mental diseases. They provide literature references to substantiate these and show that they reproduce in both the US and Swedish data.

The paper presents original research. Although DST time shift have been researched before, no one have done it this systematically for as many different diseases as here. The statistical method seems to be a standard Bayesian approach without any novelties in it. With a minor exception it seems rigorous and sound. The main novelties are that they include a wide range of diseases in one study and use a larger population than used in earlier studies of DST effect on health. For example, they state that the DST effect on pregnancy diagnoses have never been studied before. The article has relevance both for the scientific community and for decision makers who are arguing over stopping DST. It contains conclusions only possible due to the large data set. It is my impression that the paper should be published with minor revision. However, I have a number of questions, which I would like the authors to address.

We thank reviewer for appreciating our work and for providing valuable feedback. Please find point-by-point answers to your questions below.

Regarding the calculation of relative risk: The sentence about which days are compared to which (week after DST to +/- two weeks) can be complicated to understand, and I suggest putting in a figure reference to fig S2.

We agree. In the manuscript’s revised version, we direct readers to a more detailed description of the model in the Supplementary Materials document; we have also added a reference to S2 Fig (now S1 Fig.), as suggested (see lines 96-97 in the revised manuscript).

As the authors mention as a weakness, there might be other causes that drive the difference in disease rate than DST. However, the authors have chosen not to control for other than age, and gender (and race/ethnicity?). Given the data there it is possible to control for pre-existing diseases/diagnoses in the patients, even in large-scale analysis. Or to repeat a selection of the significant results in a mores strictly controlled manner. I would like the authors comments on why they did not choose to include control for any other diseases.

We made every effort to control for all confounding factors we could think of and for which we have available data. Subjects’ age, sex, geographical locations, and day length are accounted for in our analyses. Unfortunately, information on ethnicity or race was not accessible in the administrative data sets that we used (other than in an aggregated form).

Regarding controlling for pre-existing diseases and for the recurrent diagnoses versus first incidence situation: We did apply our model to the first-time diagnoses of each patient’s temporal disease code sequence—but those signals were not significant or remarkable (see the discussion in the Supplement Materials sections 1.10 and 2.4). We do agree that, in a refined model, such as a mixed-effect regression model, we could include some chronic diseases as confounding factors to obtain better estimates of disease relative risks. We did not attempt to control for any particular disease for two reasons: (1) introducing control diseases would cause a combinatorial explosion in the model space, and; (2) controlling for specific diseases would exponentially increase the number of necessary statistical tests. Both issues increase analysis complexity, make interpretation more difficult, and decrease our ability to detect signals. In the revised manuscript, we explain this reasoning (see lines 239-246 in the revised manuscript).

A very crucial detail in the author’s work is that they made negative controls. In this way, they demonstrate that it is the DST having an effect on the results and that it is not reproducible in a random week. However, I find it highly questionable to use data from prior to 1980 as negative controls in Sweden. These numbers represent a different era of medicine than the newest data and coding practices might have changed significantly. I see their validity to demonstrate that the trend starts with introduction of DST, but not as comparison to the general numbers.

We do agree that the data before 1980 in Sweden is imperfect in terms of its size and the divergent coding criteria. We addressed this issue by applying appropriate ICD mapping (ICD-8, 9, and 10, Swedish version, see S16 Table). Rather than inconsistent coding, the major difficulty in our analysis was insufficient sample size. We did not have sufficient data to analyze every condition in every age/sex group. We alleviated the coding problem using two methods: (1) careful ICD version mapping (S14-S16 Tables), and; (2) considering relatively large constellations of diseases instead of following very specific conditions (for example, we analyze “ischemic heart diseases,” including acute myocardial infarction, instead of specifically “acute myocardial infarction”). We feel these categories of larger disease groups are robust enough to withstand changes in medical practice and diagnosis criteria.

We modified our revised manuscript to better articulate this important point (see lines 283-294).

The data contains coding in both ICD versions 8, 9, and 10. There are very big differences going from version 9 to 10. Have the authors investigated if the coding frequency change abruptly because of the change? And have they verified in any way that this does not affect their results.

The coding frequency did seem to change significantly over time. We observed, particularly in the Swedish data but not as much in the US data, an abrupt change in many disease incidence rates during the transition between ICD versions. We also observed a general trend of increased incident rates in most diseases during the past decade. This observed trend could be due to an actual increase in disease incidence rates, increased healthcare resources, or an increased tendency to code diagnoses in EHRs; we found it difficult to tell all these factors apart. However, we tried to mitigate their influence by collapsing all the years’ records, so that the observation of incidence on a particular day before or after DST was calculated as the sum of incidences across the studied years. Our evaluated relative risk should then be interpreted as the average risk estimate across all the years we studied. Intuitively, neither the change in ICD version nor the increased incidence trend should be a problem because the RR is always the result of comparing incidences within just a few weeks when any change in ICD coding or administrative rules was probably minor.

The author’s mention inconclusive results from reference 6, 12, and 13 (p 5, l 60). What are the results? They later write that these the inconclusive results from reference 13 are resolved (p. 10 l 179-180), but does not clearly state which results were resolved and how.

References 6 and 12 describe conflicting results regarding whether the DST shift is associated with accident incidence. Reference 13 reports increased mental health- and behavioral health-oriented emergency department visits in certain seasons, but does not obtain conclusive results on whether they could be linked to the DST shift.

We clarify this point in the revised manuscript (see lines 51-55 in the revised manuscript).

Did the authors have any way of distinguishing between acute and planned/follow-up visits? It seems likely that the acute visits are more affected by the DST than visits planned from before. E.g. for the injuries mentioned p. 10 l 193-194, where planned visits are probably not related to the DST. Could this change the result of the analysis?

Due to the intrinsic incompleteness of claim sequences for all the patients in our data, we have no way to determine whether visits were acute or pre-planned. However, we did try our best to perform a first-time diagnosis analysis in the US data—our attempts failed to discover any significant or remarkable signals (see the discussion Supplement Materials sections 1.10 and 2.4). We were unable to analyze a complete version of the Swedish registry (due to the fact that the one we used is in-patient only), so we did not attempt a first-time diagnosis analysis for all visits on the Swedish data.

The results show a change of childbirth RR (p 8 l 146). If the number of childbirth changes, so does the number of complications related to childbirths. Could this not alone explain the other maternal codes (p 8 l 144)? I.e. does the rate of complications increase more than the observed increase in birthrate?

The change in absolute birthrate is a plausible explanation for the increase in the complications of maternal health and delivery, but we did not see any significant change in birthrates in our data across the weeks around the DST shift. We introduce this point in the revised manuscript (see lines 220-222).

In page 10 l 90 the authors mention that chronic diseases were probably preexisting. How was this investigated? There is no documentation for this statement, which should be easy to produce (i.e. number of cases where the chronic disease was seen before). Was such analysis carried out for other diseases?

In the data, we observed repeated chronic condition diagnostic codes for many patients. As expected, the most common chronic diseases were cardiovascular and metabolic, such as hypertension, arrhythmia, and diabetes. Nevertheless, a subset of conditions is clearly acute. For example, a heart attack diagnosis is unlikely to hide a follow-up appointment, and this is the assumption we used to interpret such codes in our analysis.

In supplementary section 2.4 (p 15), the significant first-time signal is dismissed as “likely to be due to seasonal convexity or concavity”. I assume that the numbers are calculated with the same +/- 14 days comparison and that this handles the seasonal convexity or concavity. How come that the main analysis does not have the same problem?

In a different study (currently submitted elsewhere), we specifically looked at disease seasonality. The most drastic seasonality effects are observed for infections; for infections, the corresponding prevalence trends change sharply over short intervals (think of almost delta-function-like spikes in seasonal influenza), violating the assumption of short-term quasi-linearity of trends required for our analysis.

Our correction, seen in equation (8) and the Supplementary Materials page 5, works properly for the majority of conditions that follow the general annual diagnosis trend we estimated for all diseases jointly, but it may not be reliable for infections.

It is not clear what the goal of the geographic location comparison is, if it can a priori be dismissed because many other covariates could influence the signal.

We admit that the results may not be really useful, but we retain them for the completeness of analysis.

The authors present three sets of results with Bayesian, “half Bayesian” and frequentist methods. All the comparison is in the supplementary methods. Can they provide more information on the advantages / disadvantages for these methods in the main section?

We have added more details regarding the differences between and advantages for each method in the main text (see lines 114-124).

I unfortunately do not know a lot about Bayesian statistics, and therefore I am not able to assess the choice of models. In the interest of the uninformed reader, I would ask that the authors explain their method in a way more readable to people with less knowledge about Bayesian statistics. I give examples of what I would like to be clearer here.

It is not clear at all how the actual data comes in to the model. In frequentist statistics, you estimate the parameters by maximizing the likelihood function. The authors give a formula to calculate the diagnosis rate on the time point of interest from the RR (5). But is the RR not the value you want to estimate and p0 a value you can calculate from the data? It would help understanding if the authors made more clear what loss function is being minimized / maximized to estimates the parameters.

In the interest of understanding the flow of the model, I don't understand how the x* (6a-6c) affects the model. They are not mentioned in the formulas for estimating the RR, but depends on the p* values.

In the revised version of the Supplementary Materials, we explain the Bayesian estimation methodology in more detail. Basically, the Bayesian procedure involves a generative model that requires a step-by-step data “construction” mechanism, based the on stochastic process defined by the model.

Based on the model and the observed data, we could compute the likelihood, and the posterior distribution could be inferred by the Bayes theorem:

posterior ∝ prior * likelihood.

We applied the MCMC procedure to approximate the intractable integral and obtain a better estimation of the posterior distribution gradually. The biggest difference between the frequentist method and a Bayesian procedure is that frequentists usually deal with the likelihood, but Bayesians try to approximate the “posterior distribution” of an estimate though a “sampler” (MCMC), please also see reply to the previous question and lines 114-124 in the main text.

The authors correct for the number of enrolled patients in the US data. As far as I understand they compare data points within +/- 2 week from the DST shift. Is the change in enrollment really so large that you have to correct for difference over a few weeks? Could they give a number on how much it changes on average? Or is it to correct for the yearly difference? In the latter case, it can be argued that the Swedish population also has changed during the study period.

We have added a new plot (see S15 Fig in the Supplement) to show enrollee variation in the US data. Integrating the models’ real-time enrollee number is an easy step to account for the confounding enrollment variation. Around DST shifts, the total enrollee number does not change much (around one percent on average, as seen in the plot below), but the yearly difference is significant. We observe that the number of enrollees changes several folds (yearly) from 2003 to 2014. The majority of these changes occur at the beginning of each year when new enrollees either joined or left the insurance policy. However, these yearly changes would not significantly affect the RR estimation, as we always compared the incidences from the few weeks around the DST shift. The Swedish national registry data did not contain enrollment information, as patients are automatically enrolled from birth to death (unless they leave the country). This means the Swedish data is likely to be more stable. We would still expect changes around the DST shift, but they are not likely to be large enough to influence our estimates in any major way.

Regarding the Bayesian correction for multiple testing, I don't understand how it works. I would suggest that the authors give more background to explain this. As I understand, the method forces the RR values towards the mean (i.e. 1 or neutral value?) when there is insufficient data. This makes a lot of sense. However, it is not clear how this shrinking is affected by the number of RR values that are estimated. And it is not clear how you can set the different thresholds for the US and Swedish data. Also, I find that the sentence regarding the shrinking in the main method-overview section should be extended to explain.

In the revised main text, we try to better explain how the Bayesian multiple test correction works – we highly recommend carefully reading the original paper by Gelman *et al.* (see the reference below), which explains the concept better than we can do here given space limitations. Essentially, there are no multiple comparisons in Bayesian analysis because we put all the RR estimation processes into one all-inclusive model, which pushes all our estimates to one or the mean (shrinking), instead of estimating each parameter separately. The joint estimation allows us to regulate how conservative (forced to be close to one) the estimates are, given the amount of observable data.

We set a different threshold for the Swedish data set because it was about five times smaller than the US data set. Our main concern was that we wanted to induce more signals given limited data. We argue that 99 percent is already a stringent threshold.

Gelman A, Hill J, Yajima M. Why We (Usually) Don’t Have to Worry About Multiple Comparisons. J Res Educ Eff. Informa UK Limited; 2012;5: 189–211. doi:10.1080/19345747.2011.618213

I assume that all the author’s choices of priors are well thought off and perhaps even standard choices. It would help if they cite some method papers that informs the reader about these choices.

Indeed, our prior choices are standard in Bayesian statistics. They are either non-informative, like Beta(1,1) (uniform distribution), or weakly-informative such as HalfCauchy(5). Please see <https://github.com/stan-dev/stan/wiki/Prior-Choice-Recommendations> for more information about the rationale for our prior choices.

The need to create the corrected RR value is well argued. However, I do not understand how the expected RR ( E[RR] hat ) is calculated and how this solves the problem. Is the expected RR the distribution's mean across all RR estimates that naturally have the temporal change in RR factored in?

E[RR] hat is exactly the posterior estimate of the Gamma distribution's mean across all RR estimates. This estimate naturally gives the average curvature across all diseases, so it could be used to account for the general convex trend seen around the DST shifts. Equivalently, we could just initialize mean = 1 in Expression 3 to enforce our prior belief that DST would not influence disease incidence.

Also, there are no comment on choice of parameters for the optimization. Are there any non-standard choices that might be relevant for readers with knowledge about such models?

We have added more details about the MCMC estimation procedure. All optimization parameters are the standard and default choices of PyMC3. Also, we diagnosed every RR estimate’s convergence (mixing) by using the Gelman-Rubin statistic (see the supplementary tables, where the Gelman-Rubin statistics are supplied for every RR). All the Gelman-Rubin statistics we computed were very close to one, which indicates that the MCMC trace mixed well (meaning, it sampled parameter values in a representative manner rather than being stuck in some area).

The typesetting of Supplementary Materials is off: There are several unintended space, some of the letters also almost overlap, and the line spacing is also varying much.
Examples
Suppl Materials page 3, section 1.1 Mappings
All ICD-10 codes are categorized i n this grouping, so i t i s also exhaustive.

Suppl Materials page 4 btw formula (4) and (4a)
"mean and l et i nformation flow [...] comparisons problem i s alleviated

We apologize for the typesetting bugs. We performed an additional round of proofreading.

**Reviewer #2**

The study by Zhang et al presents a phenome-wide study of the effects of daylight savings time and disease incidence. The study is interesting, and it is encouring to see results for both men and women. Some of the results piques my interest, although I would like to see a more thorough dissemination of the results. I was also disappointed to not see a more through comparison of the two cohorts in the main text. Surely the advantage of working with multiple cohorts must be to replicate the findings and/or perform a meta analysis. I also have some concerns with the study design, statistical analysis, and terminology throughout the article. I find it very positive that the authors have provided all code and data in a Github Repo. I also see that the authors actually report some absolute risk differences. I think this would be much more intuitive, compared to the ratios used throughout the article.

We thank reviewer for providing valuable feedback. Please find point-by-point answers to your concerns below.

INTRODUCTION

The introduction reads nicely, and provides a timely summary of the subject. One minor thing is I believe the appropriate terminology for this kind of analysis is "natural experiment" and not "large-scale intervention experiment". The authors note that previous studies typically focus on a small number of diseases. There is actually a perfectly good reason for this, which I will get back to.

The reviewer is correct. The project is not an intervention experiment but a natural exposure test. We have changed the wording from “large-scale intervention experiment” to the more appropriate “natural exposure experiment.”

METHODS

The description of the two data sources seem incomplete. Surely not only the Swedish inpatient registry was used. This would not contain information on death/emigration.

We added more details regarding the enrollment (see Supplementary S15 Fig), follow-up time(see Supplementary S16 Fig) of the US MarketScan database.

L72: "visibility" is typically called "under observation" in epidemiological jargon. Likewise, subjects are "censored" when they are not under observation. It would be very beneficial to provide average/mean follow-up time for each individual.

We have changed the word “visible” to “under observation” as the reviewer suggested. The distribution of the follow-up time is now supplied for reference (see the plot above). The mean follow-up time is 154 weeks. There are spikes for multiples of year or month (like 52 weeks, 104 weeks, etc.) because people tended to enroll in multiples of years or months.

L72: In all fairness, how are these patients guarantied to be unique? Are they linked through a social security number similar to Sweden?

In the US MarketScan database, the patients are uniquely represented by personalized, unique ID numbers (original social security numbers are used by the provider to link raw records for each subject, although these identifiable pieces of information are not retained in the sanitized dataset).

L75-76: unclear why individuals that are dead or have emigrated are excluded. This is very atypical. Please provide clarification.

It seems that there is some misunderstanding. No subject was excluded intentionally from analysis. Some of the people described in the data died or emigrated and became invisible to the registry. We mentioned this to highlight the stability and completeness of the Swedish national data, not the other way around.

From the Data description it is not clear which date is used for the patients. The admission or discharge date?

We used the dates of diagnosis.

L85: It is not clear at all how the incidence is calculated. If incidence is calculated as #cases / population at risk, the age ranges are far too wide in my opinion. Also, are cases from the previous year considered in the next year? Or are they completely excluded from the calculation? This can also be seen in the Results section, where women aged 41-60 have an increased incidence of maternal care codes. The vast majority of this group of women will not be at risk to even be pregnant. This goes back to the point as to why previous studies typically deal with a limited number of conditions: defining the population at risk is not trivial, and it is something the authors need to consider in more detail for each condition. Just using the full population will severely deflate and provide nonsense incidence estimates.

The disease diagnosis rate (or, incidence rate) is calculated as the ratio of the number of cases diagnosed during that particular day or week and the number of people enrolled during that particular day or week.

Given this definition, cases from the previous year are not considered, and all disease incidence cases are counted only once.

We defined the age range using the following consideration: A too-narrow age range would result in zero-inflated counts of relatively rare events (making estimates noisy). Therefore, we selected wider age brackets, but, arguably, not absurdly wide. Note that we aimed at selecting a set of consistent, pre-defined age bins for all diseases, for the purpose of cross-phenome scanning.

We agree that some specific diseases carry an additional risk for older age groups. We estimated the RR within the same age group by comparing the incidences of the same age group around the DST shift. Therefore, we argue that the higher RR estimates we obtained for 41 to 60-year old women presumably reflect the true risk increase, linked to the DST shift.

L95-98: I would really like to see a correction for year as well.

We corrected for year-specificity in two ways: (1) by collapsing incidences across all years of study (Expressions 7a and 7b), and; (2) by modeling means. We manually curated the DST shift dates, as well as all holidays, to fit every year’s difference, ensuring they were the actual dates of interest. We computed all diagnosis rates for a particular time point (day or week) by precisely considering the actual number of diagnoses and enrollees at that time point. In this way, the change of enrollment, diagnosis criterion, or any other factors that change throughout the study years would not affect the RR estimation. We did perform an additional analysis to account for the effect-of-the-year by introducing a year-specific random effect. However, this addition to the model made MCMC sampling unstable, indicating the more complicated model’s poor fit. The WAIC (Watanabe–Akaike information criterion) did demonstrate the poor model specification after adding another layer to correct the effect of year. Before adding the year effect layer, the WAIC is 7.93e4, but it grew about ten times to 7.19e5 after accounting for the effect of each year. We saw similar results when using the LOO (leave-one-out cross validation) statistic, which is 8.30e4 versus 7.54e5 for original and year-specific models, respectively.

L98-101: The specification of the Bayesian model is poorly explained. Please provide more detail. Additionally, I am not sure what kind of pooling the authors are using. The main text suggests a complete pooling, but the Supplementary Material looks like a no pooling scheme to me. The multiple comparison problem is not necessarily solved by just specifying priors. If, however, the authors had used a partial pooling scheme across diagnoses, this would indeed solve the multiple comparison problem. See e.g. the 8 schools example by Gelman and Gelman's Bayesian Data Analysis, 3rd ed page 101. Given that the authors work in the ICD-10 space it should be possible to make use of that hierarchy in a model with partial pooling across diagnoses.

Apologies for not making this point clearer in our original supplementary methods section: All RR estimates (for all disease groups) were sampled simultaneously within the same inference framework; individual estimates constrained each other within one hierarchical model. We explained this point carefully in the revised methods section.

How was the convergence of the Bayesian model assessed? From the github repository, it seems that the authors used PyMC3. PyMC3, to my knowledge, uses the HMC-NUTS that provides a lot of diagnostics. Please also add a citation to PyMC3 in the Main Text.

As requested, we have added more details about our MCMC estimation procedure. All the optimization parameters that we used in our analysis are the standard and default choices of PyMC3 (we have modified the description to clarify this issue). Every RR estimate’s convergence (mixing) was diagnosed using the Gelman-Rubin statistic (see the supplementary tables, in which the Gelman-Rubin statistic value is supplied against each RR estimate). We noticed that all Gelman-Rubin statistics were very close to one, indicating that the MCMC process mixed well. Also, as requested, we have added a citation to the PyMC3 in the revised version of supplementary materials (reference 47).

L100: The use of a conservative prior is good, but the prior specifications in the Supplementary Material need a lot more justification. Also, the use is non-informative priors is generally not recommended. The authors should use priors that put more emphasis on the null value if that is the prior belief.

All prior choices in our analysis follow the Bayesian statistical standard. They are either non-informative, like Beta(1,1), or weakly-informative, like HalfCauchy(5). Please see <https://github.com/stan-dev/stan/wiki/Prior-Choice-Recommendations> for more information about the rationale for our prior choices. Gelman recommended the HalfCauchy prior as it showed good performance in the sampling procedure--better than an absolute, non-informative, uniform prior. Another, more informative choice is setting the mean in Expression 3 to 1. We experimented with various priors, and the results are pretty stable to prior choice. Therefore, we decided to put another flexible modeling layer over the Gamma’s parameters and let the data “speak for itself”.

L113: by "not observing", do the authors mean that these states do not have DST?

Yes. We meant that these states did not implement DST.

L121: when was the decision made to analyze inpatient only? And in which data set?

For the US data, we originally conducted an analysis on all visits and realized that the results might be affected by many sociological confounding factors. We then decided to add another analysis using inpatient visits, only. We presented and discussed the results from both types of analysis in the manuscript.

For the Swedish data, we had access to the inpatient data, only. Thus, we performed an inpatient analysis only.

L124: I disagree with the notion of urgent problems in inpatient care. What I believe the authors mean is that there are a different kind of diagnosis associated with each encounter. E.g. pneumonia, sexually transmitted diseases, etc may be handled in outpatient or GP care. Likewise, routine surgeries represent a fairly low risk and non-acute inpatient group.

Agreed. As suggested, we have rephrased this statement to stress the intrinsic difference between inpatient and outpatient diagnoses. Some diseases, such as heart attack, are more likely to be diagnosed as an inpatient. Other diseases are, indeed, more likely to be documented during outpatient visits (see lines 139-142).

RESULTS

L128-129: Could the author write how many, or refer to a table? just refering to "a number" seems very imprecise.

The exact numbers for the detected signals are supplied in S19 Table in the Supplementary Materials. Following the reviewer’s recommendation, we have revised the main text to guide readers to the corresponding table.

L131: What are stand-out increases? Do the author mean relative large effect sizes?

We meant that they are both evidential (meaning, they are in the top 30 in terms of the effect size) and of interest to us for further discussion.

L134-135: "Signal" - would that be the change in relative risk? What is "automatically detected according to their effect size"?

We have changed the word “signal,” to the phrase, “signals of relative risk change.” The phrase “automatically selected according to their effect size” means that the presented signals were selected impartially via a computational procedure designed to avoid a cherry-picking bias. This selection procedure is described in the Section 2.1 of the Supplementary Materials.

L140: This is unclear. Did the point estimate of the frequentist confidence interval increase after FCR correction? Or do the authors mean that the frequentist point estimate is greater (in absolute terms), compared to the Bayesian analysis? The latter is expected due to the shrinkage and should not come as a surprise to the authors.

The reviewer is correct. The effect size estimated by the frequentist method tends to be larger because estimates were not shrunk as were those obtained with the Bayesian partial pooling method.

L142-143: I am not convinced about this result due to the lack of a proper at-risk group. The group at-risk should, in this case, be pregnant women, and not just all women.

We estimated the RR within the same age group by comparing to incidences of the

same age group around the DST shift, so the high RR we gave for the 41-60 years-old

women were presumably extra risk associated with the DST shift. Ideally, it would be more judicious to customize experiment groups for every disease we test.

L159: Is it to be understood that the only signal that replicates are the cardiovascular diseases under a less stringent threshold? This is very disappointing, and in my opinion this strongly indicates that there is a bias in the data set that confers these increases that is unrelated to DST. A population of 9 million individuals is not a small data set, and it should have plenty of power to detect differences in incidence for single diseases.

In general, it would be good to see the all results simultaneously to see if there are trends within disease areas, using e.g. the ICD-10 chapter structure.

We have provided the processed incidence numbers and numbers of enrollees for the US MarketScan data on the GitHub web site. Sharing this information will allow others to replicate our study very easily and permit them to explore trends within the disease areas. For the Swedish national registry, we are not allowed to reveal the number of incidences.

We were unable to replicate all the results in the Swedish data. This is most likely due to two reasons: (1) The Swedish data is about five times smaller than the MarketScan data and inpatient-only, and; (2) Signals observed in the US analyses are more vulnerable to heterogeneity (data produced by over 100 insurance companies with diverse practice norms). Though the Swedish national registries are more homogeneous and complete, they are smaller. We have added more discussion about the replication problem in the Limitation section.

DISCUSSION

L179-180: not clear what exactly is meant here. What is the previous result, and why was it inconclusive?

Reference 13 observed increased mental and behavioral health emergency department visits in certain seasons but did not obtain conclusive results on whether they could be linked to DST shifts. We clarify this point in the revised manuscript, please see our replies to Reviewer 1.

L184: was this result presented previous? A _ten_ percent increase in cardiovascular and heart disease in a population of children and teenagers seems very extreme. How many children actually had a cardiovascular disease? An absolute risk would give more insight into the actual consequence.

The absolute risk of posed by the DST shift is shown in S28-31 Tables and discussed in Section 2.5 of the Supplementary Materials. We estimate that about 17 additional cases per year of “other forms of heart disease” among females in the 0 to10 years-old population occur in the week following the spring DST shift and 20 additional cases of “other forms of heart disease” among the males 10 to 20 years-old population (S30 Table). For this particular disease, the absolute numbers are not very large.

L184-185: I am not a big fan of presenting both Bayesian and Frequest estimates jointly. The Bayesian model that the authors have specified naturally shrinks the estimates towards the null, so it is no surprise that point estimate is smaller than the frequentist procedure. This also indicates, strongly, that there is not a lot of data in support of the values, and that the prior has a strong influence on the estimate.

The reviewer is correct. The large difference between the Bayesian estimates and the frequentist estimates highlight the inadequacy of the data. We have rephrased our statement about the method comparison (see lines 205-208).

L189-190: Could these groups very well be the individuals that are covered by an insurance plan? I would imagine children and teenagers are covered by their parents insurance plan.

Yes. In the IBM MarketScan database, children and teenagers are usually recorded as dependents of their parents or guardians.

L195: What does "emergency care" refer to here? Were they admitted to the ER?

We meant only that these diseases were acute and required immediate care, such as heart attacks. We have changed the phrase to “immediate care.”

L198-199: without any previous studies exploring this, this is pure unfounded speculation and should be stated as such.

We agree that this is speculation. We have rephrased this statement in a more prudent way and suggested it is but one possible explanation.

L202: This is not guaranteed. Insured individuals have a completely different socioeconomic profile versus the uninsured, and extrapolating between the two requires information on the uninsured. This is a general concern when using insurance data, namely that it is highly biased towards specific socioeconomic profiles. This could be explored in the Swedish data set, as there should be registries containing information regarding employment for these dates.

Agreed; we have removed the statement that uninsured people are likely to be affected by DST shifts at least as much as the insured population. We did not have the employment information ready for analysis.

L210-215: I am surprised to see this analysis done in the US data set, and not the Swedish data set, as this would cover more age groups since there is complete follow-up since 1977. I.e. it would be possible to follow an individual for up-to 37years.

The Swedish dataset is much smaller than the US, and it is limited to inpatient events. For this reason, we restricted our analysis to the larger US data.

L216-220: Or, alternatively, in some years there has been pandemics around the DST. This would not be the case if the authors had adjusted for calendar year.

It is not clear how correcting for a specific year could be implemented in our model. One possible way is to treat the RR as a value that varies every year, and follow a Student’s-t distribution with a central mean RR. The pandemics could then be captured as outlying confounder factors.

L231-232: This is false. The low p-values / narrow intervals are driven by the large data set. This does not mean that the data set is free of bias, and any bias will propagate and can very well drive associations. This is ultimately the cause as to why replication and meta analysis are so vital, and it would improve the article immensely if the authors did this.

We have changed this discussion about the reported effects as requested (see lines 262-264).

L240: Do the Swedish data set actually record the exact diagnosis date, and not just the dates of the inpatient stay?

Yes. They record the actual diagnoses dates.

L246: That is an interesting point. How was the sex of each patient actually identified? Could an alternative explanation not also be that some people change sex?

Both the US and Swedish data provided patients’ sexes. We used this information to estimate the error-coding rate (see Section 1.12 of the Supplementary Materials). We estimated the error rate to be at about 0.52 percent. Sex changes, ambiguous sexuality, intersex, etc. are alternative explanations. However, we argue they are likely to be less prevalent than the observed 0.52 percent.

According to

M. Lane, G. C. Ives, E. C. Sluiter, J. F. Waljee, T. H. Yao, H. M. Hu, W. M. Kuzon. Trends in Gender-affirming Surgery in Insured Patients in the United States. Plast Reconstr Surg Glob Open. 2018;6(4):e1738.

“An estimated 0.6 percent of the US population identifies as transgender and an increasing number of patients are presenting for gender-related medical and surgical services.” About 1/7 of these is followed by a gender-affirming surgery.

L252: If one is to compare the miscoding rate to the observed effects, it would be better to estimate the miscoding rate per diagnosis. Giving the overall rate does not provide a lot of information, since some diagnosis are far more frequent than others.

Agreed. However, we did not have an obvious approach to gauge the per-diagnosis error rate. The 0.52 percent rate we give is a general rough reference of the errors that could occur in the data source.

**Reviewer #3**

In this manuscript the authors demonstrate measurable health effects that are associated with the Daylight Saving Time Shift.
Using two databases, USA claims and Sweden's inpatient register, the authors found known (in literature) associations in heart diseases and injuries and also identified some novel associations related to maternal problems related to pregnancy, mental and behavioral disorders, immune-related conditions. This is a very innovative use of patient data to provide data and numbers to the debate whether DST shifts are healthy or unhealthy for populations. These associations are quite interesting and with further study can uncover additional relevant health effects associated to DST changes. Making this work highly relevant in the field.

The study design is quite clear and the methodology is exhaustively detailed within the manuscript and the abundant supplemental materials, code is available for replication. However, replicability is still not directly possible as the underlying data sources are not available (commercial and protected data) and the pre-processing of said data sources is not fully detailed or available either. The literature review is quite sufficient highlighting relevant papers to the posed problem and the methods used. While the main manuscript details are good, the real meat of this article is on the supplemental materials. The use of positive and well defined negative controls is rigorous and adequate for this type of study.

We thank reviewer for appreciating the work and providing us with valuable feedback. Please find point-by-point answers to your questions below.

One issue that is not mentioned is that sometimes claims and EHR data is anonymized in a way that patient timelines are date-shifted. This would greatly impact any results that have to do with temporality of patient diagnoses. While I don't believe that Marketscan is time shifted, I believe is something that should be addressed.

There are some databases that anonymize the data by shifting the time stamps. We did consult the manager of the MarketScan database and the managers of the Swedish national registry to ensure that the time stamps upon which we rely are accurate and meaningful.

The signals found on the Swedish data are quite few, and it is unclear that if the relaxation they did (99.9 vs 99 CI) actually allowed them to find more (table S19 shows the Swedish signals found this way?), Finding 7 significant associations leaves plenty of the ones found in the USA data unmatched an non evaluated(?). This makes a case to potentially use a third data source for sanity.

We set a different significance threshold for the Swedish data because it is about five times smaller than the US data. The main concern is that we wanted to induce more signals with this limited data. We argue that 99 percent credible interval is already acceptably stringent. We also found that most signals were not replicable in the Swedish data even after we relaxed the threshold. Thus, we encourage people to be prudent and ponder the findings. In the revised manuscript, we have added a new paragraph to the Limitation section which frames a more conservative approach to the replication problem.

On potential improvement of this work is the user of a standardized common data model, such as OMOP or pcornet. This would also allow the same study to the executed at other places with data converted to the common data model and the associations could be easily validated, without the need to share any data. This would greatly enhance the impact of the contribution and provide a clear way of validating the findings.

We have provided the processed incidence numbers and enrollees numbers for the US MarketScan data on the GitHub web site (https://github.com/hanxinzhang/dst/tree/master/data). Sharing this information will allow others to easily replicate our study and permit them to explore trends within the disease areas. For the Swedish national registry, we are not allowed to reveal the number of incidences.

Minor details:

Supplemental Materials (S1 Appendix) figure 1 is extremely hard to read, with the confidence intervals not clearly showing.

We apologize for the extremely small size of the violin plots in Figure 1. We have provided the S1 Fig (now S2 Fig) separately for more clear reference. We produced the figure in vector-graphics PDF format; it can be magnified in a viewer application for closer detail. The 99.9 percent C.I. is shown on each violin using vertical bars.
