# Supplementary material for: Measurable health effects associated with the daylight saving time shift
Source: PLoS Comput Biol. 2020 Jun 8;16(6):e1007927. doi: 10.1371/journal.pcbi.1007927 (PMC7302868; doi:10.1371/journal.pcbi.1007927)
Supplement: Attachment — Submitted filename: response to reviewers_DST_AR.docx [file pcbi.1007927.s033.docx]

Reviewer #1: The authors have addressed all my mentioned concerns except one very minor issue:
They cited the prior choice recommendation in the answer to my and another reviewer. However, they chose not to include this. I would encourage the authors to cite it.
[https://github.com/stan-dev/stan/wiki/Prior-Choice-Recommendations](https://urldefense.proofpoint.com/v2/url?u=https-3A__github.com_stan-2Ddev_stan_wiki_Prior-2DChoice-2DRecommendations&d=DwQGaQ&c=Nd1gv_ZWYNIRyZYZmXb18oVfc3lTqv2smA_esABG70U&r=ULvNIgo15mH_8cCBmiM1KBF_qHRW8ZMYO-_ZDPm3uOp9kFqARW63OFcx12Y06DIX&m=dOXO-IvLFdcHeX_7qW6d6PgAn2bey5VknRVZX26y7tk&s=EC7Nleo4rDjyQZ1cVg29XRKMtoTnG3UAPeYY_S4IEFU&e=)
I trust that this can be fixed without further input from my side.

Thank you for reminding us to include this reference. We have included the citation in the new revision.

Reviewer #2: I would like to commend the authors on doing a good job on clarifying uncertainties, which has led to an improvement of the manuscript. I have a few remaining comments,

The mean follow-up time for the US MarketScan database is 154, or just shy of 3 years. I think this would be valuable to add to the Methods section, as this would heavily reflect the denominator.

We added the mean follow-up time abnalysis in the Methods section of the new revision.

Regarding the date of diagnosis, I am not sure I follow. In a 2011 description of the Swedish Inpatient Registry, the variables listed are "Admission date" and "Discharge date" ([https://www.ncbi.nlm.nih.gov/pmc/articles/PMC3142234/](https://urldefense.proofpoint.com/v2/url?u=https-3A__www.ncbi.nlm.nih.gov_pmc_articles_PMC3142234_&d=DwQGaQ&c=Nd1gv_ZWYNIRyZYZmXb18oVfc3lTqv2smA_esABG70U&r=ULvNIgo15mH_8cCBmiM1KBF_qHRW8ZMYO-_ZDPm3uOp9kFqARW63OFcx12Y06DIX&m=dOXO-IvLFdcHeX_7qW6d6PgAn2bey5VknRVZX26y7tk&s=uNtF4k7ZfqKoqGEXWhzQvLlZHB8fiVqtuwOgiUVH6YQ&e=)). Typically, the date is validated on a per-disease basis, and for spinal cord injury there was only a 62% agreement. There is no "Date of diagnosis", as far as I can see. Could the authors please clarify this? Additionally, could the authors also comment on how an inaccuracy of the diagnosis date would reflect their results?

We apologize for the imprecise description. We added discussion about relationship between possible inaccuracy of disease diagnosis dates and interpretation of our results.

We used “discharge dates.” Such dates may or may not be close to the dates of diagnosis, considering that the physician’s final advice and prescription would be given in the discharge summary. In most cases, neither the date of diagnosis as in the US data nor the discharge date is expected to be the same as the actual time of disease onset. But, because chronologically billing follows clinical care, “discharge date” is typically closer to disease onset than the “billing date” is. We took into account possible noise in diagnosis dates by adding up all discharge codes in the week following the daylight-saving time change.

I am still not sure I understand the hierarchical model that the authors employ. If all RR estimates are sampled simultaneously within the same framework, does that mean that all estimates come from a gamma distribution with mean mu? If so, is this not problematic and can lead to hyper-shrinkage? Normally this would not be an issue, but in a model as this that pools together so many different outcomes (injuries, heart disease, pregnancy), is it really expected that they all have the same mu?

You are correct. We sampled all RR estimates simultaneously from the same prior distribution with the same mu to avoid the multiple comparisons problem. We use the same mean for all estimates because our prior believes are (1) most diseases are not affected by the DST change, and (2) the effects are very small (RR close to 1) for diseases that are perturbed by the DST change. We are not estimating RRs of heterogeneous characteristics. Such prior setting should be as safe as initializing a weakly-informative prior with location zero like Cauchy(location=0, scale=5), as Bayesians always do.

Hyper-shrinkage might be a valid concern when we do not have enough diagnoses for some diseases. Therefore, we considered large-scale disease classes such as “ischemic heart diseases” instead of a small disease category “acute myocardial infarction.” As we can see in S5 and S6 Figs (S5 is also attached here), though the Bayesian method shrinks the estimates to the mean, it still gives estimates of the same magnitude as the frequentist methods do. It does not overly shrink the estimates.

I also find it odd that the authors still highlight complications in maternal care despite using a seriously flawed denominator. I think the authors would see this if they counted the number of women > 45 that are pregnant in both cohorts. If the authors are serious about this, they should validate it using a carefully crafted at-risk group.

We removed our spotlight discussion on the maternal care in the new revision. We apologize that we are not able to extract the information about pregnancy and redefine our denominator in a timely manner. We decide to move such analysis to a future study focusing on more carefully crafted at-risk groups.

Reviewer #3: Thanks for addressing my concerns and sharing the incidence numbers they found on the MarketScan data.

Thank you for reviewing the manuscirpt!
